# A functional reference map of the RNF8 interactome in cancer

**DOI:** 10.1186/s13062-022-00331-z

**Published:** 2022-07-13

**Authors:** Chuanyang Liu, Jingyu Kuang, Yuxuan Wang, Ting Duan, Lu Min, Chenyu Lu, Tianyi Zhang, Ruifen Chen, Ying Wu, Lingyun Zhu

**Affiliations:** 1 Department of Biology and Chemistry, College of Sciences, National University of Defense Technology, Changsha, 410073 Hunan China; 2 Key Laboratory of Elemene Class Anti-Cancer Chinese Medicine of Zhejiang Province, Hangzhou Normal University, Hangzhou, 311121 Zhejiang China; 3 Joint Logistic Support Force 921th Hospital, Changsha, 410073 Hunan China; 4 Department of Critical Care Medicine, Second Xiangya Hospital, Central South University, Changsha, People’s Republic of China

**Keywords:** Pancancer analysis, RNF8, Interactome, YBX1, Ubiquitination

## Abstract

**Background:**

RNF8 is an E3 ligase identified as a critical DNA damage-responsive protein. Recently, multiple reports have shown that RNF8 could be used as an important therapeutic target for cancer chemo/radiotherapy. However, the understanding of RNF8 remains limited due to the lack of its interactome reference map and comprehensive analysis of RNF8 in diverse cancers, which underscores the need to map the interactome of RNF8 via high-throughput methods.

**Results:**

A two-way identification method based on LC–MS was designed for the identification of the RNF8 interactome with high-specificity. By in silico analysis and in vitro validation, we identified a new reference map of the RNF8 interactome network containing many new targets, such as YBX1, DNMT1, and HDCA1, new biological functions and the gene-disease associations of RNF8. Our results revealed a close relationship between RNF8 and neurodegenerative diseases or tumor-infiltrating immune cells using bulk RNA-seq and scRNA-seq datasets. As a proof of concept of our interactome map, we validated the direct binding between RNF8 and YBX1 and showed that RNF8 catalyzed the ubiquitination of YBX1. These results demonstrated that RNF8 might be a crucial regulator of YBX1.

**Conclusions:**

Our work provides a unique framework for researchers and clinicians who seek to better explore or understand RNF8-regulated biological functions in cancers. This study will hopefully facilitate the rational design and further development of anti-RNF8 therapy in cancers.

**Graphical abstract:**

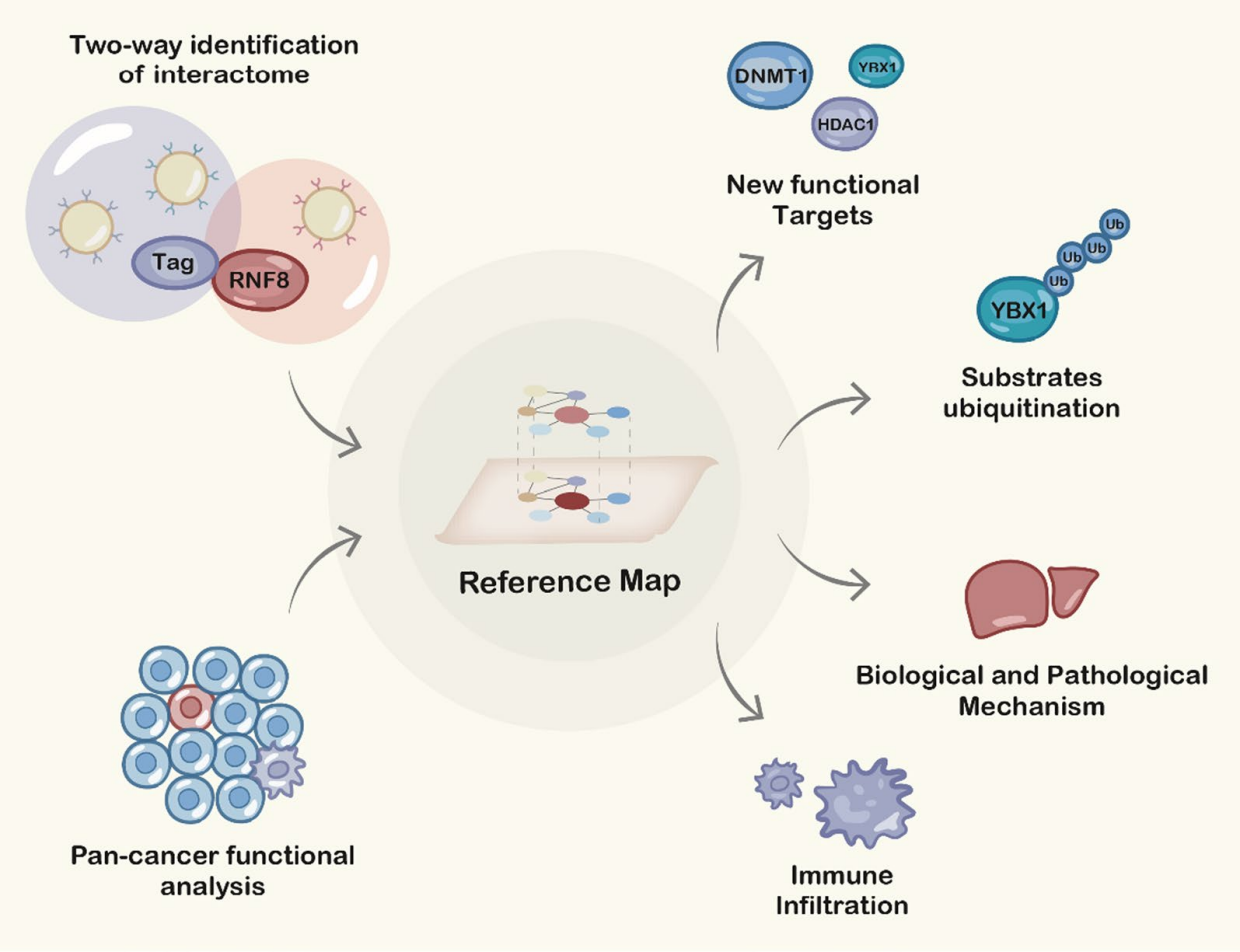

**Supplementary Information:**

The online version contains supplementary material available at 10.1186/s13062-022-00331-z.

## Background

RNF8 (UniProtKB Accession Number: O76064) is a 484-amino-acid E3 ligase that consists of two conserved domains [1, 2]: an N-terminal forkhead-associated (FHA) domain, a phosphopeptide recognition domain found in many regulatory proteins, and C-terminal Really Interesting New Gene (RING) domain that catalyze the formation of ubiquitin chains on substrates, which is responsible for its E3 ubiquitin ligase activity [3]. Together with E1 ubiquitin-activating enzyme and E2 ubiquitin-conjugating enzymes such as Ubc13, RNF8 is capable of catalyzing the assembly of K63-/K48-linked polyubiquitin chains on its substrates and therefore contributes to their nucleic translocation and protein degradation [4]. RNF8 was originally identified as a critical DNA damage-responsive protein and contributes importantly to DNA double-strand repair (DSB) repair. In response to DSBs induced by stimuli such as ionizing radiation and reactive oxygen species, the C-terminus of histone H2A variant (H2AX) is phosphorylated and interacts with MDC1, which leads to the recruitment of RNF8 to the sites of DSBs via its FHA domain and therefore mediates the formation of K63-linked polyubiquitin chains on histones [3, 5]. This ubiquitin signaling promotes the subsequent recruitment of multiple DNA damage repair factors such as 53BP1 and BRCA1, thereby enabling homologous directed repair. In addition, RNF8 targets nonhomologous end joining factor KU (KU70/KU80) for K48-linked ubiquitination [6], and promotes efficient DSB damage repair by decreasing the proapoptotic activity of p53 through regulating Tip60 protein activity [7].

In addition to DNA double-strand break repair, RNF8 has also been found to play important roles in various biological processes [7, 8], such as chromatin remodeling [9], inflammation signaling [10], spermatogenesis [1, 11], telomere maintenance, and end protection [12], by interacting with divergent target proteins. Therefore, RNF8 can be partly recognized as the “Guardian” of our cells. However, even though many gain-of-function and loss-of-function studies have elucidated the distinct function of RNF8 in divergent physiological statuses, the general function of RNF8 has been poorly discussed due to the lack of high-throughput identification of its interactome (or substrates), which underscores the need to comprehensively reveal the molecular mechanism underlying this crucial biological process.

Recently, several findings revealed oncogenic potential might be the other side of the coin of RNF8 functions. Studies have shown that RNF8 is also involved in many cancer-associated biological processes such as tumorigenesis and cancer metastasis. Kuang et al. found that RNF8 promotes breast cancer epithelial–mesenchymal transition (EMT), an essential process in cancer metastasis that facilitates the infiltration of tumor cells into surrounding tissues [13, 14], via inactivation of GSK-3β and activation of β-catenin signaling [3]. A similar breast cancer-promoting rule of RNF8 was also demonstrated by Lee et al., which showed that RNF8 facilitates cancer chemoresistance and progression through activation of Twist, a transcriptional control factor of the epithelial–mesenchymal transition, by RNF8-mediated K63-linked polyubiquitin [4, 15]. The critical roles of RNF8 in lung cancer tumorigenesis [2, 16], bladder cancer radiosensitivity [17], hepatocellular carcinoma growth, and metastasis [18] were also revealed recently. In our previous study, we also revealed that RNF8 promoted epithelial–mesenchymal transition in lung cancer [2]. Despite the emerging understanding of the biological function of RNF8 in cancers, the underlying molecular mechanism of how RNF8 and its interactome network regulate divergent pathways and phenotypes is needed.

Here, we integrated liquid chromatography-tandem mass spectrometry (LC–MS) and massive bioinformatic analyses to profile the interactome and overall functions of RNF8. The aim of this study was to systematically reveal the potential molecular mechanism underlying RNF8-associated biological processes and establish a comprehensive RNF8-interactome regulation network with high specificity, which might reinforce the association between RNF8 and its regulated physiological and pathological processes. These findings will provide a reference map for exploring, understanding and analyzing RNF8-related biological processes and clinical value.

## Results

### Pancancer expression pattern and prognostic value of RNF8

**Fig. 1:**
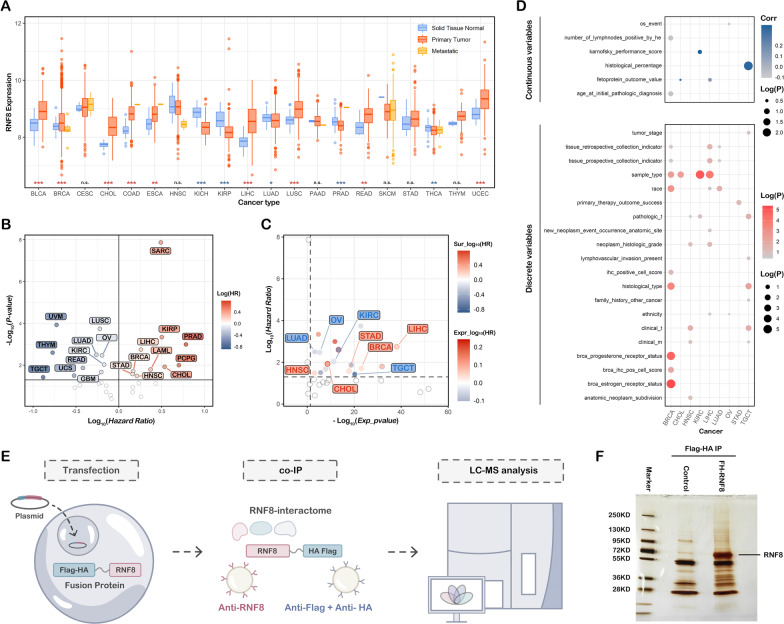
Pancancer expression pattern and prognostic value of RNF8. **A** The Pancancer normalized tissue samples were downloaded from UCSC Pan-cancer data hub. The expression of RNF8 in cancers was visualized by R package “ggplot2” and Wilcoxon rank-sum test was performed to compare the mean between cancer tissues and normal tissues. **B** Kaplan–Meier survival analysis was performed based on cancer types, The R package “survival” and “survminer” were used and the optimal cutoff of RNF8 expression was obtained using function “surv_cutpoint”. The data in each cancer was collected and visualized in scatterplot using Log_10_(Hazard Ratio) as the x-axis and − Log_10_(*P* value) as the y-axis. **C** Pancancer expression data and survival results were collected and visualized in scatterplot using Log_10_(*P* value-survival) as the y-axis and − Log_10_(*P* value-expression) as the y-axis. **D** Heatmap showed the correlation between RNF8 expression and clinical parameters. **E** Graphical representation of the workflow in identifying RNF8-interacting proteins. The RNF8-interacting proteins complexes were obtained by two different co-IP methods (co-IP using anti-Flag antibody, the elution was then used to perform another round of co-IP using anti-HA antibody; co-IP using anti-RNF8 antibody), SDS-PAGE gel electrophoresis and silver staining were performed to select the specific band. **F** The specific bands were sent to liquid-mass spectrometry (LC–MS) for analysis. **P < 0.01, ***P < 0.001

First, to reveal the expression pattern in different cancer types, the expression of RNF8 was analyzed in pancancer tissues and associated normal tissues. The results showed that the expression of RNF8 is significantly upregulated in bladder urothelial carcinoma (BLCA), breast invasive carcinoma (BRCA), cholangiocarcinoma (CHOL), colon adenocarcinoma (COAD), esophageal carcinoma (ESCA), liver hepatocellular carcinoma (LIHC), lung squamous cell carcinoma (LUSC), rectum adenocarcinoma (READ) and uterine corpus endometrial carcinoma (UCEC). In addition, significant downregulation of RNF8 was observed in kidney chromophobe (KICH), kidney renal papillary cell carcinoma (KIRP), lung adenocarcinoma (LUAD), prostate adenocarcinoma (PRAD) and thyroid carcinoma (THCA) (Fig. 1A, Additional file 1: Fig. S1A and Additional file 1: Fig. S1B). Then, Kaplan–Meier analysis was performed to assess the potential prognostic value of RNF8 in cancers, and the results are shown in Fig. 1B.

Integrating the aberrant expression pattern with the prognostic value, we identified nine cancer types that were strongly associated with tumorigenesis and prognosis: LIHC, STAD, BRCA, CHOL, HNSC, LUAD, OV, KIRC and TCGT. In LICH, STAD, BRCA, CHOL and HNSC (Group 1), elevated expression of RNF8 was observed in tumor tissues and was related to poor prognosis. In LUAD, OV, KIRC and TCGT (Group 2), the trend was reversed. These results indicated that, among these nine cancers, RNF8 expression might be a crucial factor for tumorigenesis and cancer progression. Therefore, the correlation between RNF8 expression and clinical parameters of cancers was further analyzed, and the results showed that RNF8 was significantly associated with parameters such as sample type, pathologic_t and neoplasm grade in Group 1 (Fig. 1D). In breast cancer, RNF8 is associated with many key clinical parameters such as estrogen receptor status, IHC positive cells, and progesterone receptor. Therefore, a further elaborate analysis was performed and is shown in Additional file 1: Fig. S1C and Additional file 2: Fig. S2. In Group 2 cancers, the expression of RNF8 was associated with distinct clinical parameters when compared to Group 1. Taken together, these results further demonstrated the connection between RNF8 and clinical parameters in different cancers.

### Identification of the RNF8 interactome via LC–MS

The distinct and critical roles of RNF8 in tumorigenesis, cancer progression and resistance to chemotherapy are found both in our results and many previous reports [2, 3, 19–21]. We are quite curious about how RNF8 regulates abundant and different biological processes such as breast cancer metastasis [3, 19], lung cancer tumorigenesis [16], DNA double-strand break repair [7, 8], chromatin remodeling [9] and spermatogenesis [1, 11]. Considering that the functional basis of RNF8 is basically to interact with its targets, we thought identifying its direct targets and interacting proteins is more useful to understand how RNF8 participates in various biological processes, such as LC–MS, compared to other high-throughput omics.

Therefore, we designed a high-specificity RNF8-interactome identification method by integrating anti-RNF8-based IP with anti-Flag(+ anti-HA)-based IP (Fig. 1E). Briefly, based on the transfection of the Flag-HA (FH-tag)-RNF8 plasmid, a co-IP assay using anti-Flag-M2-beads was performed to obtain the Flag-HA-RNF8 protein and its interacting proteins. To further improve the specificity of the IP assay, another round of co-IP using anti-HA-beads was performed using elution from the previous step, to obtain FH-RNF8-interacting proteins. SDS–PAGE and silver staining were performed to verify the expression of RNF8 and specific bands that could be sent to liquid-mass spectrometry (LC–MS) were chosen for analysis (Fig. 1F). In addition, another co-IP assay using an anti-RNF8 antibody was also performed to minimize the potential nonspecific interacting protein signal.

**Fig. 2:**
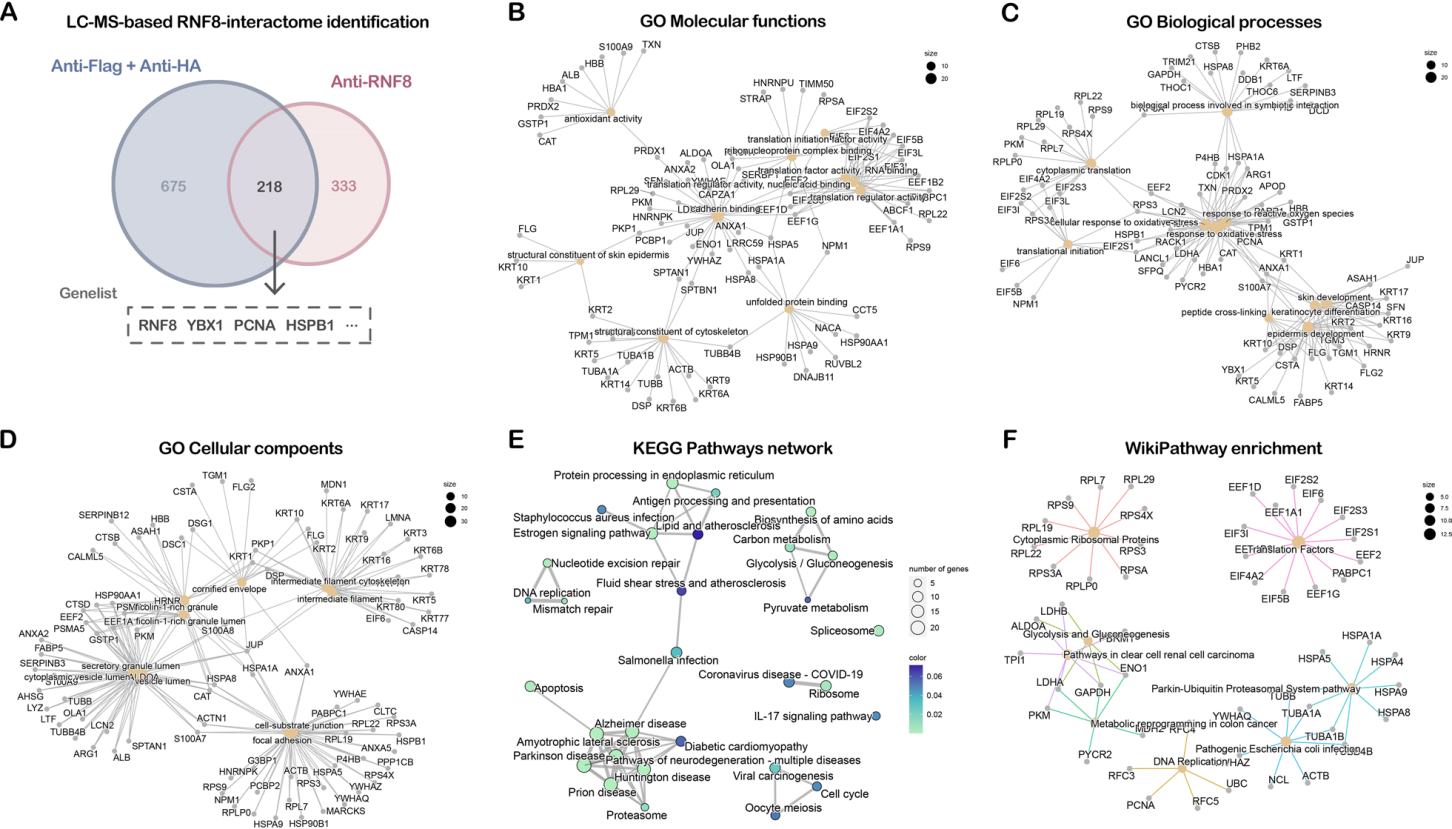
Identification and functional analysis of RNF8-interacting proteins. **A** The RNF8-interacting proteins complexes were obtained by two different co-IP assays (co-IP using anti-Flag antibody, the elution was then used to perform another round of co-IP using anti-HA antibody; co-IP using anti-RNF8 antibody). Gene ontology (GO) annotation analysis, KEGG pathway, and WikiPathway enrichment analysis were performed and visualized using R package “clusterprofiler” version 4.0. **B** GO molecular functions annotation, **C** GO biological processes annotation. **D** GO cellular components annotation. **E** Network connection of enriched KEGG pathways. **F** WikiPathway enrichment. The top-8 enriched items were shown in the network plot

Two RNF8 interactomes (893 proteins in the anti-FH group and 551 proteins in the anti-RNF8 group) were acquired via LC–MS (Fig. 2A, Additional file 15: Table S1). The protein IDs in the two datasets were converted to Entrez IDs, and the intersection of these two protein sets was integrated and visualized. Finally, 218 overlapping proteins, including YBX1 and PCNA were obtained (Fig. 2C and Additional file 15: Table S1). Together, coupling IP assay-based assays and Venn diagram-based filtering resulted in a comprehensive RNF8-interacting protein list with high specificity, allowing downstream integrative analysis.

### Functional enrichment of the RNF8 interactome

To further understand the functional and regulatory patterns of the RNF8 interactome at the cellular level, we performed GO annotation and KEGG pathway enrichment using the functional enrichment package clusterProfiler [22]. As shown in Fig. 2B, terms such as cell adhesion molecular binding, translation factor activity, RNA binding, and structural constituent of cytoskeleton were enriched in GO molecular functions (MFs). Regarding GO biological processes (BPs), these 218 proteins were mainly enriched in epidermal cell differentiation, keratinocyte differentiation, neutrophil-mediated immunity, neutrophil activation, etc. (Fig. 2C). Regarding cellular components (CCs), the proteins were significantly enriched in the cell-substrate junction, focal adhesion, cytoplasmic vesicle lumen, and intermediate filament cytoskeleton (Fig. 2D). Furthermore, neurodegenerative diseases such as the Parkinson's disease, Alzheimer's disease, and amyotrophic lateral sclerosis were enriched by the KEGG pathway (Fig. 2E). Additionally, protein processing in the endoplasmic reticulum, DNA replication and repair, coronavirus disease (COVID-19), and the estrogen signaling pathway were enriched. For Wikipathway enrichment, the most enriched terms were cytoplasmic ribosomal proteins, translational factors, glycolysis and gluconeogenesis, DNA replication, and the parkin-ubiquitin proteasomal system pathway (Fig. 2F). These results indicate that there might be unrecognized associations between RNF8 and these processes.

Other important biological pathways were also shown with identified target proteins (Table 1). To our surprise, apart from DNA repair, the p53 signaling pathway, the cell cycle, chromatin organization and the TNF-α pathway, which have been partly reported in RNF8-related studies [23], many of the identified targets have not yet been validated. In addition, the correlation between RNF8 and many of the identified pathways such as oncogenic MAPK pathways, epigenetic regulation, TGF-beta receptor, sumoylation, integumentary system disease, keratosis, skin disease (Additional file 3: Fig. S3A and B), and most of the identified target proteins, has not been reported, which underscores deeper exploration and more mechanistic insights toward RNF8.

**Table 1:** The enriched key pathways and identified targets

Pathway	Members
Epigenetic regulation of gene expression	HDAC1; SIRT1; UBTF; CHD4; GTF2H3; DNMT1; RBBP4; POLR2E; POLR1A; DEK; MBD3; MYBBP1A; GTF2H4; MTA2
Chromatin organization	HDAC1; KDM1A; WDR5; RUVBL1; CHD4; HCFC1; MTA2; NFKB2; RUVBL2; PRMT5; MORF4L1; ACTL6A; MBD3; PBRM1; SMARCA2; RBBP4; MORF4L2; WDR77; KDM5A
DNA repair	PCNA; POLR2E; DDB1; TP53; RAD51; GTF2H3; TCEA1; POLR2A; COPS5; APEX1; CDK2; PARP1; TP53BP1; ACTL6A; POLR2C; ASCC3; RUVBL1; GTF2H4; XRCC5; XRCC6
TNFalpha	HDAC1; TXN; DDX3X; LRPPRC; POLR1A; MCM7; YWHAB; MAPK1; ACTL6A; CDK9; CCNT1; FLNA; PSMC2; NFKB2
Oncogenic MAPK signaling	PHB; MACF1; FXR1; MAPK1; SND1; YWHAB; MEF2C; PPP2CA
TGF_beta_Receptor	HDAC1; DAXX; TP53; COPS5; MEF2C; CDK2; MAPK1; EWSR1
C-MYC pathway	PPP2CA; RUVBL1; ACTL6A; RUVBL2
SUMOylation	PCNA; TP53; NUP98; PARP1; HNRNPK; TP53BP1; HNRNPC; RNF2
p53 signaling pathway	PCNA; CDK2; TP53
mRNA splicing	SRSF1; SRSF2; POLR2E; POLR2A; POLR2C; YBX1; PRPF6……
Cell cycle	HDAC1; AHCTF1; RUVBL1; TP53; RUVBL2; PPP2CA; NUP98; RBBP4; MCM7; PCNA; CDK2; MAPK1; MCM3; TERF2IP; YWHAB; TP53BP1; NPM1

### Construction and clustering of the PPI network

To further elucidate the potential interactions among the 218 overlapping proteins, a protein–protein network was constructed utilizing the Metascape database [22] with the following databases: STRING^6^, BioGrid^7^, OmniPath^8^ and InWeb_IM^9^. Only physical interactions in STRING (physical score > 0.132) and BioGrid were used. The resultant network contains the subset of proteins that form physical interactions with at least one other member in the list. Then, the Molecular Complex Detection (MCODE) algorithm^10^ was applied to identify densely connected network components (Additional file 4: Fig. S4A), which is biased in favor of showing the characteristics of the network (Additional file 4: Fig. S4B–F).

Further functional analysis showed the direct relationship between identified proteins and biological processes such as proteasome, nucleotide excision repair (MCODE Cluster 1), target ribosome, RNA transport (MCODE Cluster 2), pathogenic Escherichia coli infection, phagesome (MCODE Cluster 3), spliceosome, Alzheimer’s disease (MCODE Cluster 4) and oocyte meiosis (MCODE Cluster 5). These results further reinforced the relationship between RNF8’s target proteins in these clusters and corresponding diseases or biological processes, which might also be the unrecognized functions of RNF8 (Additional file 5: Fig. S5).

### The correlation between RNF8 and biological pathways in cancers

**Fig. 3:**
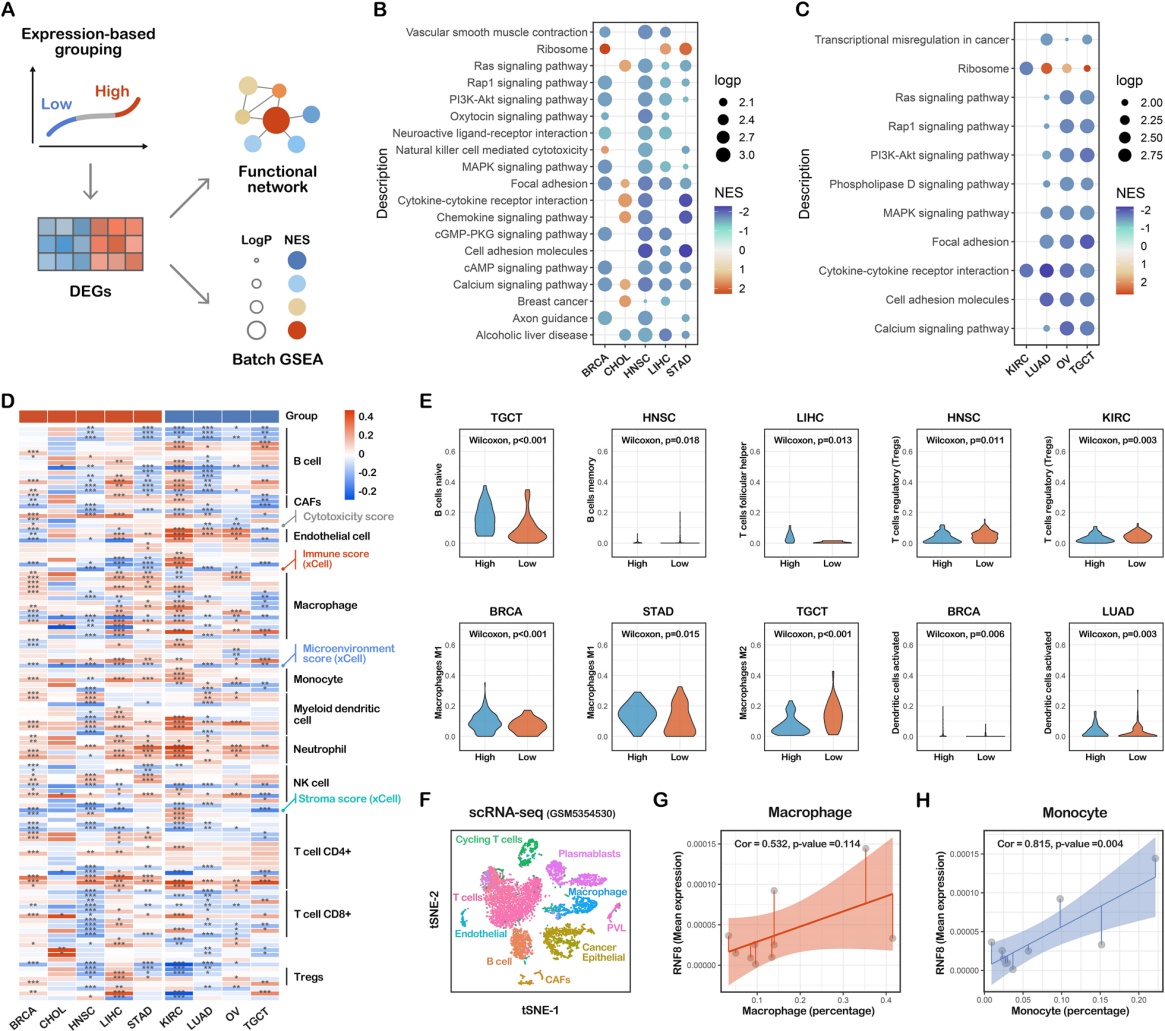
The correlation between RNF8 and tumor-infiltrating immune cells. **A** Schematic representation of expression-based grouping method. Pancancer-normalized expression dataset were trisected based on RNF8 expression in different cancers. Differential gene expression analysis of RNF8 high-expression group compared to low-expression group and subsequent functional analysis including batch Gene set enrichment analysis were used to identify functional profile of RNF8 (**B**, **C**). Batch Gene set enrichment analysis reveal the general functions in cancers. **D** Six immune cell infiltration algorithms including Cibersort, xCell, EPIC, MCP-counter, quanTIseq, TIMER,were utilized to calculate the immune cells-infiltration score in pancancer dataset. Scores of different immune cells were clustered and visualized as heatmap. **E** violin plot showing the immune cells scores in different cancer. **F** tSNE dimension reducation were visualized based on single cell transcriptome data (GSM5354530) of breast cancer tissues. **G**, **H** Ten single cell transcriptome data were analyzed using R package Seurat to reveal the correlation between RNF8 expression (the mean expression of RNF8 in all cells). and the immune cell percentage (in non-tumor cells) in BRCA tissues. tSNE dimension reduction were performed and cell type annotation were match to each cell

To further reduce the bias in the identification of the RNF8 functional profile, another round of functional profiling using large-sample high-throughput sequencing data was performed (Fig. 3A). Based on pancancer-normalized mRNA abundance data of nine cancers, we trisected samples (Additional file 6: Fig. S6J–R), and divided them into an RNF8 high-expression group and an RNF8-low expression group in different cancer types. Using differential gene expression analysis between the two groups (Additional file 6: Fig. S6A–I), we identified RNF8 functions in a novel abundance-based way.

**Fig. 4:**
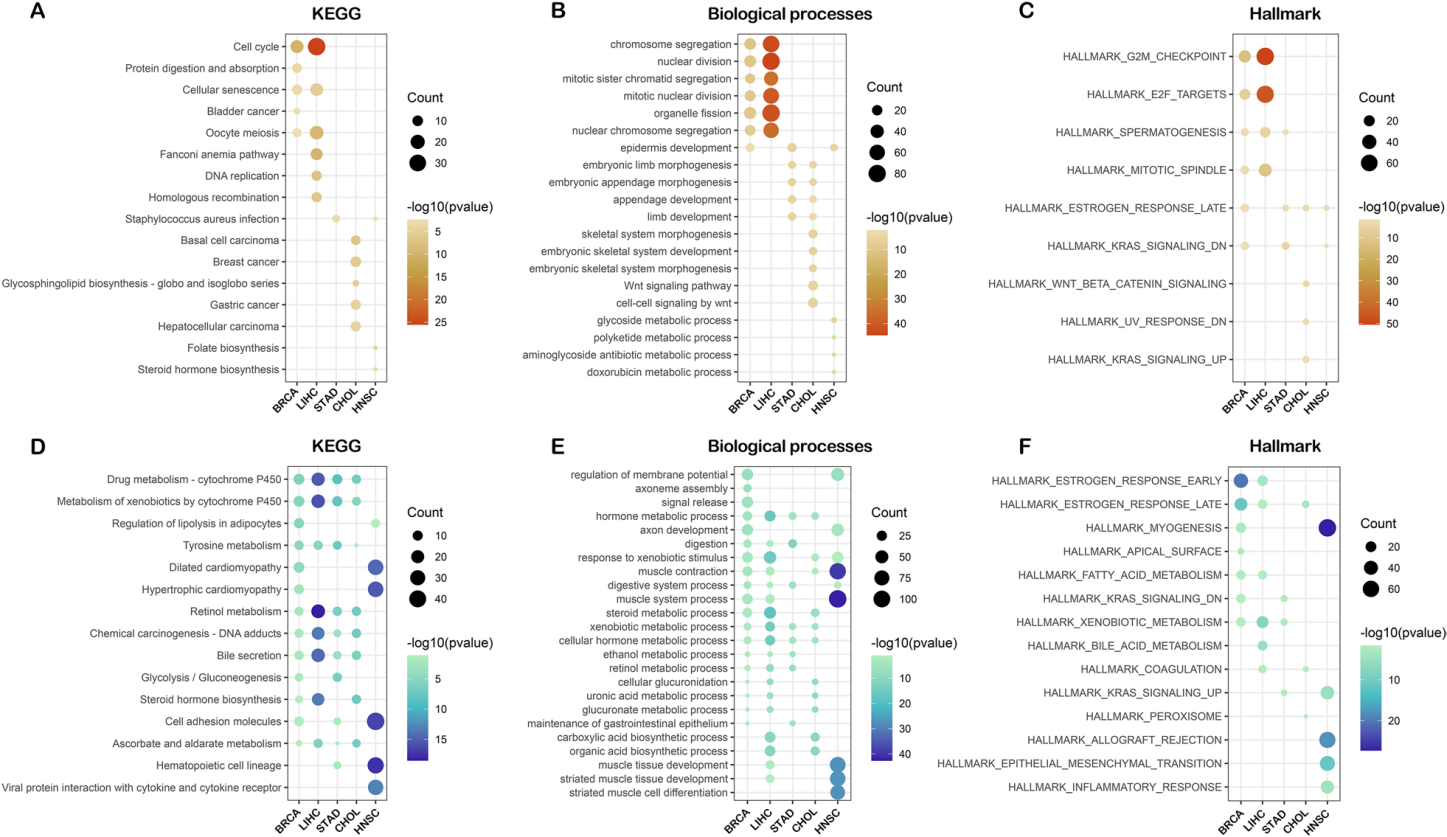
Functional analysis of RNF8 in Group1 cancers. KEGG (**A**), GO biological processes (**B**) and Hallmark (**C**) enrichment analysis were performed using identified up-regulated genes in Group1 cancers. For down-regulated genes, KEGG (**D**), GO biological processes (**E**) and Hallmark (**F**) enrichment analysis were also performed

Consistent with previous findings, gene ontology and KEGG enrichment analyses showed that the upregulated genes in Group 1 cancers were enriched in cell cycle and spermatogenesis (Fig. 4A–C, Additional file 7: Fig. S7A), while the downregulated genes were enriched in cytochrome P450 (metabolism of drug/xenobiotics), hormone metabolic process, downregulation of KRAS signaling and estrogen response (Fig. 4D–F, Additional file 7: Fig. S7B). In Group 2 cancers, however, the results showed a different picture, and the terms in which upregulated genes were enriched in did not show a common pattern (Additional file 8: Fig. S8A–D). The downregulated genes were enriched in cytokine-cytokine receptor interactions, upregulation of KRAS signaling, etc. (Additional file 8: Fig. S8E–H). In addition, batch gene set enrichment analysis showed that RNF8 was enriched in terms such as the MAPK signaling pathway, focal adhesion, and transcriptional misregulation in cancer.

### The correlation between RNF8 and biological pathways in cancers

To our surprise, we observed that immune cell-related terms such as natural killer cell mediated cytotoxicity (Fig. 3B, C), inflammatory response (Fig. 4F) and T-cell activation (Additional file 8: Fig. S8F and H) were enriched both in GSEA and multiple previous enrichment analyses, which attracted our interest. Multiple previous studies have demonstrated that RNF8 regulates the progression and metastasis of cancer cells in different cancers [2–4]. Whether RNF8 regulates tumor-infiltrating immune cells and whether this regulation affects cancer status, such as progression or metastasis are unknown.

To explore the function of RNF8 in the tumor microenvironment (TME), a six-tumor microenvironment algorithm was used to assess the abundance of cell types in nine cancers. The results showed that the correlation between RNF8 and tumor infiltration varied in different cancers (Fig. 3D). The expression-based method was utilized for further validation (Fig. 3E), In the RNF8 high expression group, significant increase in naïve B cells was observed in TGCT, as well as decreased memory B cells in HNSC, upregulated T follicular helper cells in LIHC, downregulated Tregs in HNSC and KIRC, and upregulated M1 macrophages in BRCA and STAD.

**Fig. 5:**
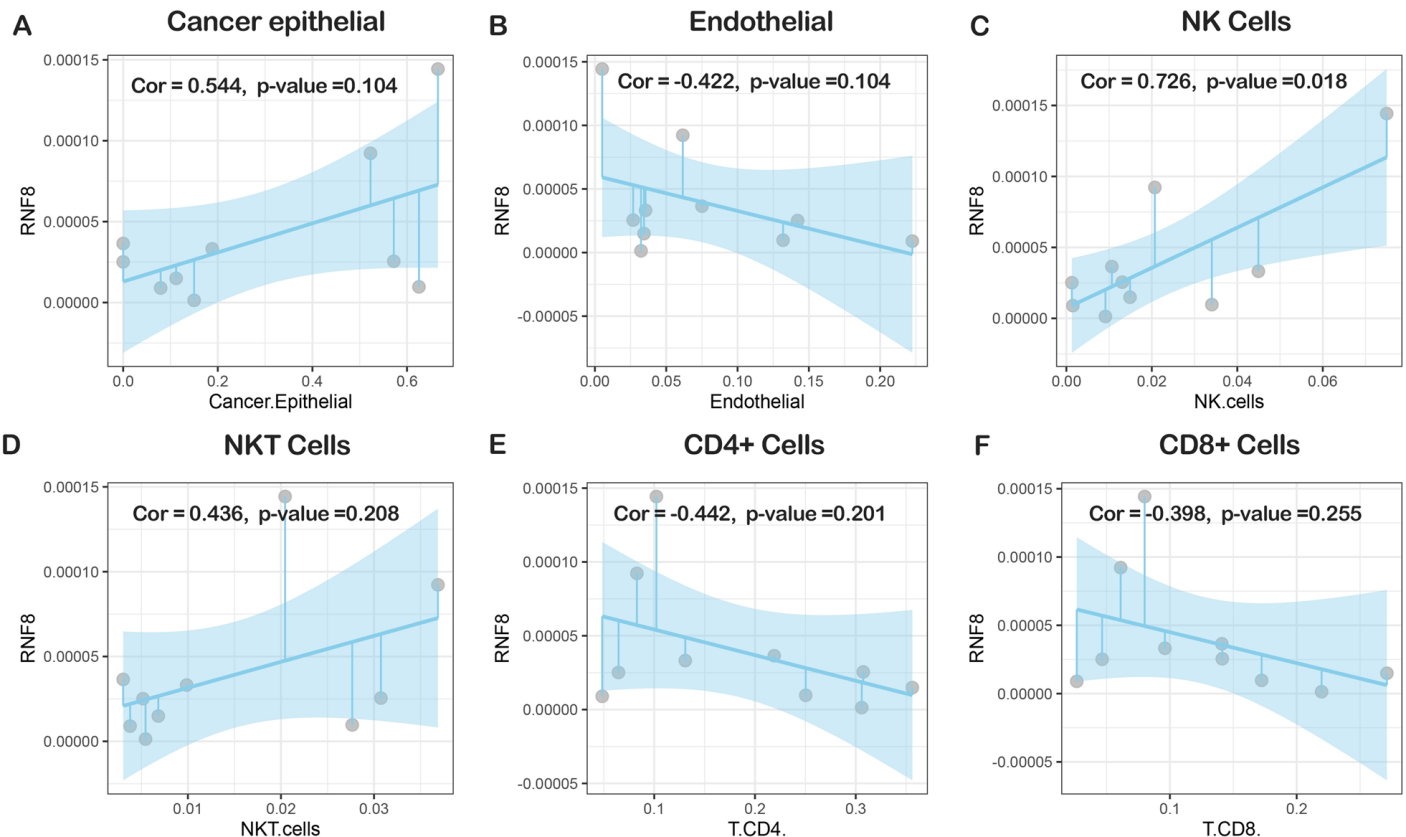
Integrated scRNA-seq analyses showing the correlation between RNF8 expression and percentage of tumor-infiltrating immune cells. **A** Cancer epithelial cells, **B** endothelial cells, **C** natural killer cells, **D** natural Killer T cell, **E** CD4+ Cell, **F** CD8+ Cell

To further validate these findings, ten TNBC breast cancer scRNA-seq datasets were collected and analyzed (Fig. 3F). The results showed that RNF8 mean expression was positively correlated with the percentage of macrophages, monocytes (Fig. 3G, H) and NK cells (Fig. 5C) among nontumorous cells, which is consistent with previous findings. In other identified immune cells, there were no significant correlations (Fig. 5).

Taken together, these findings revealed the potential correlation between RNF8 and tumor-infiltrating immune cells, which might provide new insight into RNF8’s functional heterogeneity in divergent cancers.

### Searching for potential RNF8-interacting proteins

As another way to find potential RNF8-interacting proteins, five well-known protein–protein interaction databases and prediction tools, STRING, GeneMANIA, InBio_Discover, BioGRID, and HitPredict, were screened and analyzed. As shown in Additional file 9: Fig. S9, 11 proteins appeared in at least four datasets. These proteins might be promising interacting proteins of RNF8 (Additional file 16: Table S2). Pancancer survival analysis found that these proteins, together with identified target proteins, were negatively correlated with patient survival (Additional file 10: Fig. S10 in ACC, LIHC, LUAD, SARC, showing their potential to be biomarkers for prognosis. However, when we compared these proteins with the protein list acquired by LC–MS, only a few proteins (7 in 218) were in the union of all five protein sets, which is quite surprising.

### Validation of RNF8-YBX1 interaction and functions: a case of proof-of-concept

**Fig. 6:**
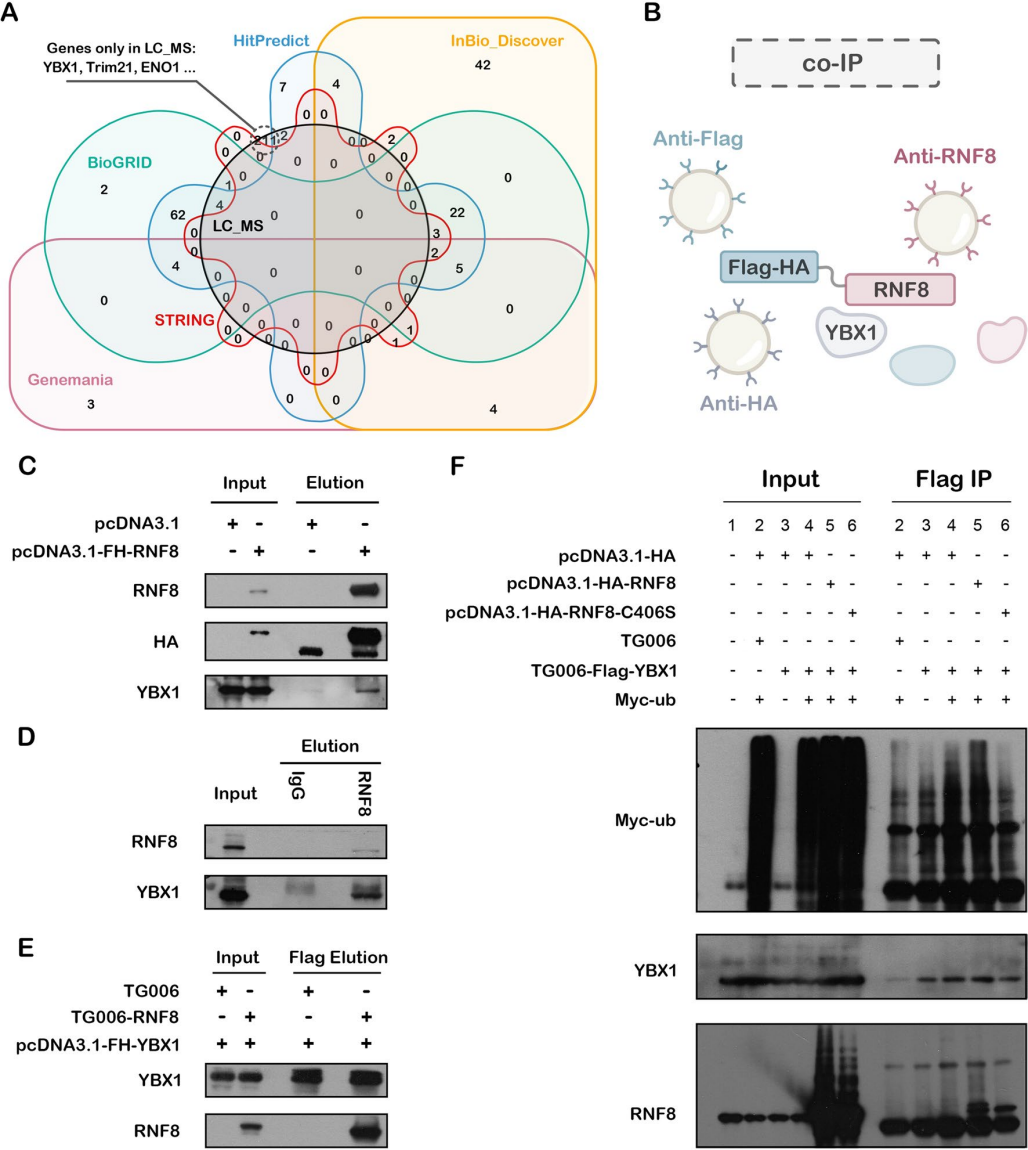
In vitro validation of the interaction between RNF8 and YBX1. **A** The intersection of RNF8 interactome by LC–MS and potential RNF8-interacting proteins acquired using five online tools. **B** Schematic diagram of Co-IP. **C** HEK-293T cells were transfected with pcDNA3.1 (control) or pcDNA3.1-FH-RNF8 and harvested after 48 h of transfection. HEK-293T cell lysates were incubated with HA-Sepharose beads, and the bound proteins were analyzed via western bolting with anti-RNF8, anti-HA, and anti-YBX1 antibodies. **D** we harvested MDA-MB-231 cells, then the lysates were co-immunoprecipitated with anti-RNF8 antibody or normal mouse IgG(control), and the elution protein complexes were analyzed by western blotting with anti-RNF8 and anti-YBX1 antibodies. **E** HEK-293T cells were transfected with TG006 or TG006-RNF8 with pcDNA3.1-FH-YBX1. At 48 h post-transfection, the cells were harvested and co-immunoprecipitated with anti-Flag-M2 agarose beads, and the YBX1 protein complexes were analyzed by western blotting with anti-YBX1 and anti-RNF8 antibodies. **F** HEK-293T cells were serially transfected with the corresponding plasmid. At 72 h post-transfection, the cells were harvested and coimmunoprecipitated with anti-Flag-M2 agarose beads, and the YBX1 protein complexes were analyzed by western blotting with anti-Myc (Ub), anti-YBX1, and anti-RNF8 antibodies

To confirm the LC–MS-identified RNF8 interactome, we chose YBX1, a critical regulator of transcription and translation that is widely recognized as an oncogenic driver in several solid tumors such as breast cancer [24, 25], which was only identified by LC–MS and not in 5 PPI databases (Fig. 6A) as the proof-of-concept target.

Three co-IP assays were designed to verify the direct interaction between RNF8 and YBX1 (Fig. 6B). As shown in Fig. 3C, the RNF8 band was observed both in the input and elution of HEK 293T cells transfected with pcDNA3.1-FH-RNF8, while in elution, YBX1 was only observed in cells transfected with pcDNA3.1-FH-RNF8, which demonstrated the direct interaction between RNF8 and YBX1. Co-IP using anti-RNF8 antibodies to pull down the endogenous RNF8 complexes showed similar results (Fig. 6D). YBX1 also pulled down RNF8 protein using anti-Flag-M2 beads (Fig. 6E), further demonstrating the direct interaction between RNF8 and YBX1.

The classic function of RNF8 is to catalyze the ubiquitination of substrates and lead to their degradation, activation, or other alterations [12, 16, 26]. To further demonstrate the mechanism underlying the interaction between RNF8 and YBX1, the ubiquitination status of YBX1 in RNF8 overexpressing or RNF8-C406S (loss-of-function mutant, attenuating the activity of catalyzing the formation of the polyubiquitin chain to substrates) cells was assessed. The results showed that overexpression of RNF8 elevated the ubiquitination of YBX1, while the introduction of the C406S mutation to RNF8 reversed the ubiquitination status of YBX1. These results further prove the interactions between RNF8 and YBX1 and demonstrate that this interaction might regulate the ubiquitination of YBX1 (Fig. 6F).

## Discussion

RNF8 is a 484-amino-acid E3 ligase located on chromosome 6p21.3 [1]. As demonstrated previously, the biological function of RNF8 largely originates from its two conserved domains: the FHA domain in the N-terminal, which binds to the phosphopeptide motif, and the RING domain in the C-terminal, which catalyzes the formation of the polyubiquitin chain [16]. As an E3 ligase, RNF8 has been shown to promote the formation of K63-, K48-, and K11-linked polyubiquitin chains when coupled with E2s such as UBC13, UBCH8, UbcH6, UBE2E3, and UBE2S, respectively. This ubiquitination of RNF8-interacting targets contributes to their nuclear translocation, activation, protein degradation, etc. [6, 27]. Based on these functions, RNF8 plays important roles in various biological processes, such as the DNA damage response, telomere protection, cell cycle control, and transcriptional regulation [5]. However, it is quite confusing that RNF8 seems to have two roles. One is that RNF8 acts as a “Guardian” of our cell; it helps transduce DNA damage signals and initiate DSB repair upon DNA damage, maintain genomic stability and participate in spermatogenesis [12]. Downmodulation of RNF8 also enhances cancer cell radiosensitivity [28]. Another role of RNF8 is to promote lung cancer tumorigenesis and chemoresistance [2, 21] and to promote breast cancer metastasis [3], similar to a “Villain”. In light of the structure of RNF8 and the way RNF8 functions, we utilized LC–MS to identify its direct targets and interacting proteins to understand how RNF8 participates in distinct biological processes.

By integrating anti-RNF8-based IP with anti-Flag(+ anti-HA)-based IP, we identified the RNF8 interactome with high specificity. Our study provides a comprehensive reference map for RNF8 functions and reveals many new potential functions of RNF8. In our results, some of the RNF8-target pairs were demonstrated in previous reports, such as RNF8-UBC [29], while most of the RNF8-target pairs were not established previously. Given the massive studies presented recently about RNF8 biological functions and potential therapeutic applications, the identification of the RNF8 interactome surely would provide substantial evidence and instructions for RNF8-related mechanistic and functional studies.

As a proof of concept, we examined the RNF8-YBX1 interaction to validate our interactome identification in vitro. YBX1 is a transcription factor that has been demonstrated to participate in the spliceosome, apoptosis, translation, cell proliferation, and tumor progression [30, 31]. Recently, YBX1 has attracted much attention. Gandhi et al. showed that lincNMR regulated tumor cell proliferation through a YBX1-RRM2-TYMS-TK1 axis governing nucleotide metabolism [32]. In addition, Goodarzi et al. found that YBX1 stabilized pro-oncogenic transcripts and enhanced cancer cell metastasis under hypoxia [33]. In our study, we demonstrated the direct interaction between RNF8 and YBX1 and found that RNF8 promotes the ubiquitination of YBX1, which provides a novel molecular mechanism underlying YBX1-related biological processes. How RNF8-mediated YBX1 ubiquitination is involved in spliceosome and tumorigenesis needs to be explored in the future.

As shown in Table 1, our results identified for the first time the direct interaction between “Genome Guardian” RNF8 and key genes in epigenetic regulation. Previous reports showed that RNF8-dependent ubiquitination of histone H2A during meiosis establishes active epigenetic modifications [34]; however, how RNF8 regulates epigenetic modification remains largely unknown. As shown in Table 1, our results suggest that RNF8 might be a potential regulator of DNMT1, a key methyltransferase that maintains the methylation status after DNA synthesis and is associated with many important biological processes, such as early embryo implantation and tumorigenesis, via direct interaction. In addition, HDCA1, a deacetylase that inactivates the expression of neuronal genes in nonnervous tissues and is implicated in axonal alteration and degeneration of the cell [35], was identified as the target of RNF8. Adam et al. showed that RNF8-dependent polyubiquitination is required for the establishment of H3K27 acetylation. RNF8 was also reported to play a role in suppressing synapse formation [29] and neuron degeneration [36], but the clear mechanism underlying these physiological processes is poorly understood. Our findings might therefore provide new clues and evidence for these biological processes.

RNF8 is regarded as a DNA damage signal transducer. Upon DNA damage, the Mre11-Rad50-Nbs1 (MRN) complex senses the damage and recruits Ataxia telangiectasia mutated (ATM) to damage sites. ATM and ATR phosphorylated histone H2AX (referred to as γH2AX), and MCD1. RNF8 is then recruited to the DNA double-strand break site through FHA domain-mediated interaction with MDC1 [37, 38] and stabilizes JMJD1C demethylase, demethylating MDC1 at K45, which promotes MDC1 association with RNF8 [39]. RNF8 then couples with Ubc13, DYRK2 and L3MBTL2 to catalyze the formation of K63-linked polyubiquitin chains on many chromatin substrates, including histones H2A, H2AX, and H1 [40, 41], which results in the recruitment of DNA repair proteins, including 53BP1, BRCA1, and RAD51, to facilitate NHEJ or HR repair. RNF8 also regulates the abundance of the nonhomologous end-joining (NHEJ) repair proteins KU80 and JMJ2A by catalyzing K48-linked polyubiquitination at sites of DNA damage and it promotes efficient DSB damage repair by decreasing the proapoptotic activity of p53 through regulating Tip60 protein activity [40]. Our results showed that RNF8 might regulate DNA repair via multiple targets such as PCNA, TP53, RAD51, and CDK2. Some of the targets are consistent with numerous reports; for example, Li demonstrated that PCNA is a target of RNF8 and is monoubiquitinated by it. However, most of the other targets and the function of RNF8-target axes have yet to be explored.

Although preliminary, we provide evidence demonstrating that RNF8 might be involved in many biological pathways. To reveal the common function of RNF8 on a larger scale, integrated bioinformatics analysis and network analysis were performed. Interestingly, in our KEGG enrichment analysis, we noticed is that RNF8 was involved in many neurodegenerative diseases, such as Parkinson's disease, Alzheimer's disease, and amyotrophic lateral sclerosis (Fig. 2D). The relationship between RNF8 and these diseases might be a new area that needs to be further explored. As shown in Siwei’s study, RNF8 deficiency results in neurodegeneration in mice [36], but how RNF8 regulates these processes and exactly how RNF8 regulates neurodegenerative diseases still need to be further explored and discussed based on the identified interactome. In addition, the role of RNF8 in immunology-related processes such as neutrophil-mediated immunity and neutrophil activation, and whether these connections affect the role of RNF8 in cancer tissues have not been discussed and require further exploration. The spliceosome is another item that is enriched in both the MCODE and KEGG pathways. Many of our results show a strong connection between RNF8 and the mRNA spliceosome. Whether RNF8 regulates spliceosome and whether the regulation occurs via YBX1 or other identified targets require further experimental validation.

We noticed that the role of RNF8 in tumorigenesis, metastasis, and chemoresistance has been increasingly reported in many cancers recently. In breast cancer, RNF8 promotes cancer progression and metastasis through Twist activation, and RNF8-mediated epithelial–mesenchymal transition is also regulated by multiple miRNAs such as miR-622 and miR-214, which is consistent with our enrichment analysis results showing that the RNF8-interactome might participate in cadherin binding and cell adhesion. With multiple newly identified RNF8 targets, our identified interactome might be an alternative mechanism underlying these RNF8-regulated biological processes [4, 19, 42]. In hepatocellular carcinoma, Trabid inhibits cancer growth and metastasis by cleaving RNF8-induced K63-linked ubiquitination of Twist [18]. RNF8 also promotes tumorigenesis in lung cancer [16], and silencing RNF8 sensitized bladder cancer to radiotherapy [17]. Our results showed that RNF8 might participate in metabolic reprogramming in colon cancer and clear cell renal cell carcinoma. Ling et al. showed that RNF8 can induce β-catenin-mediated c-Myc expression and thus promote colon cancer proliferation, which is consistent with our findings. Additionally, our identification of RNF8-interacting ENO1, LDH1, PKM, PYCR, and MDH2 might provide other mechanisms underlying RNF8-regulated colon cancer progression.

In addition, our functional analysis of RNF8 in different cancers implied that RNF8 might be associated with the function of immune cells, these data quickly attracted our interest. As discussed above, many efforts have been made to discover how RNF8 regulates cancer progression, metastasis, and prognosis, while in view of tumor microenvironment, whether RNF8 influences the physiological status of tumor-infiltrating immune cells, a class of immune cells that play critical roles in cancer progression and are closely related to clinical outcomes, is largely unknown. By analyzing bulk RNA-seq data and scRNA-seq data, our results showed a direct correlation between RNF8 expression and immune cells such as monocytes and macrophages in various cancers. These findings might provide new insight into RNF8-regulated tumor-associated biological processes and mechanisms.

In summary, our identification of the RNF8 interactome revealed numerous new targets of RNF8. Based on these identified targets and integrated bioinformatic analysis, we systematically revealed the potential functions of RNF8 at the protein–protein interaction level and pathway levels. We believe our work will provide a unique framework for researchers and clinicians who seek to better explore or understand RNF8-regulated biological functions, as well as their clinical applications.

## Conclusion

In this study, we identified a reference map of the RNF8 interactome network containing many new targets, such as YBX1, DNMT1, and HDCA1, new biological functions and the gene-disease associations of RNF8. We also revealed the unrecognized relationship between RNF8 and neurodegenerative diseases or tumor-infiltrating immune cells, and validated the direct binding between RNF8 and YBX1, and showed that RNF8 catalyzed the ubiquitination of YBX1, showing that RNF8 might be a crucial regulator of YBX1. Our work provides a unique framework for researchers and clinicians who seek to better explore or understand RNF8-regulated biological functions in cancers and diseases. This study will hopefully facilitate the rational design and further development of anti-RNF8 therapy in cancers.

## Methods

### Evaluation of the prognostic value and methylation status of RNF8

The Visualization of RNF8 methylation status, RNF8 expression, and corresponding clinical data were performed by MEXPRESS (https://mexpress.be/) [43, 44], a user-friendly online tool showing the DNA methylation, expression, and clinical data for the selected genes. The precise genomic location of DNA methylation is one of the most important regulatory factors of gene expression is also shown in MEXPRESS [45]. The TIMER 2.0 database (http://timer.cistrome.org/) is a comprehensive resource for systematical analysis of immune infiltrates across diverse cancer types [40]. The Oncomine database (https://www.oncomine.org/) is a user-friendly online tool that provides integrated analysis using datasets composed of samples represented as microarray data measuring either mRNA expression or DNA copy number on primary tumors [46].

### Searching the potential RNF8-interacting proteins

Genemania (https://genemania.org/) is an online tool that find other related genes using association data include protein and genetic interactions, pathways, co-expression, co-localization, predictions, and protein domain similarity [47]. Search Tool for the Retrieval of Interacting Genes (STRING, https://string-db.org/) [48] is a dataset to predict protein–protein interactions including physical and functional associations, deriving from computational predictions, knowledge transfer between organisms. HitPredict (http://www.hitpredict.org/) [49], BioGRID (https://thebiogrid.org/) [50] and inBio_Discover [51] (https://inbio-discover.com/#home) were also used to find the potential RNF8-interacting proteins.

### Functional enrichment analysis

Clusterprofiler, a user-friendly R package for gene annotation and analysis was utilized to make sense of one or multiple gene lists [22], for gene ontology (GO) annotation and enrichment analyses including KEGG (Kyoto Encyclopedia of Genes and Genomes) pathway and WikiPathways. Adj. *P* < 0.05 was considered significant.

### Protein–protein interaction analysis

The protein–protein interaction (PPI) network of overlapping genes was constructed by STRING [48] and Metascape [22]. In the present study, the default setting was set as the selection criterion of constructing the network, all disconnected nodes were excluded from the network. The list of PPI pairs was downloaded for further analysis and visualized by Cytoscape software (version 3.7.1). Molecular Complex Detection (MCODE) plugin in Cytoscape was utilized to find the potential cluster in the PPI network based on topology. The degree cut-off value to 2 and the node score cut-off to 0.2 were set in the MCODE process.

### Pan-cancer prognostic value of hub genes

To assess the prognostic value of hub genes in pan-cancer dataset, Gene Expression Profiling Interactive Analysis (GEPIA) tool (http://gepia.cancer-pku.cn/), including integrated TCGA mRNA sequencing data and the GTEx, were also used (with FDR *P* value adjustment, 0.05 significance level, and Median group cut-off) to calculate patient overall survival rate (OS) and relapse-free survival rate (RFS) [52, 53]. The results were shown in form of a heatmap with colors of cells showing log_10_(HR) and the frame meaning significance.

### Integrated analysis of tumor-infiltrating immune cells

Pancancer-normalized mRNA expression dataset (n = 11,060) and curated clinical data (n = 12,591) were downloaded from UCSC Xena Pan-Cancer Atlas Hub (https://pancanatlas.xenahubs.net). The samples in each cancer were analyzed separately. Samples were trisected based on RNF8 expression and divided into three group. Differential gene expression analysis was performed between RNF8 high-expression group and low-expression group. Subsequent Over-representation analysis and functional analysis including GO MF, GO BP, GO CC, HALLMARK, Reactome, Wikipathways and disease association analysis were performed using R package clusterProfiler (Ver. 4.2.1), DOSE, enrichplot and msigdbr. Identified terms with significant (*P* value < 0.05) were visualized by ggplot2.

For immune infiltrating analysis, six immune cell infiltration algorithms including Cibersort, xCell, EPIC, MCP-counter, quanTIseq, TIMER were utilized to calculate the immune cells-infiltration score in pancancer dataset using R package USCSXenaShiny [54]. Scores of different immune cells were clustered and visualized as heatmap using ggplot2.

### scRNA-seq data analysis

Ten single cell transcriptome (GSM) data were analyzed using R package Seurat. After filtering cells (nFeature_RNA > 200 & nFeature_RNA < 2500 & percent.mt < 20), expression data were normalized and scaled using “LogNormalize” method and function “ScaleData”. Uniform Manifold Approximation and Projection (UMAP) and tSNE was used for dimension reduction. With cells labelled by metadata downloaded from GEO176078. The mean expression of RNF8 in nontumorous cells were calculated as:

$${Exp}_{RNF8}=\frac{\sum_{1}^{{n}_{\mathrm{nontumorous}}}{n}_{i}{Exp}_{RNF8\_i}}{{n}_{All\, cells}-{n}_{cancer \,cells}}$$

While ${n}_{\mathrm{nontumorous}}={n}_{All\, cells}-{n}_{cancer\, cells}$. Besides, the percentage of a certain type (type x) of immune cells were calculated with following formula:

$${P}_{tumor\, infiltrating\, immune\, cells \,x}=\frac{{n}_{x}}{{n}_{\mathrm{nontumorous}}}$$

### In vitro proof of concept for bioinformatic analyses

#### Cell culture

Human embryonic kidney cell line 293T were purchased from American Type Culture Collection (ATCC, Manassas, VA), and cultured in RPMI 1640 Medium (Hyclone) supplemented with 10% fetal bovine serum (FBS; GIBCO, Gaithersburg, MD, USA) and 100 U/ml penicillin and streptomycin (P/S; Hyclone). Cells were contained in a 5% CO_2_ incubator at 37 °C.

#### Co-immunoprecipitation

For exogenous Co-IP, HEK293T or MCF7 cells were transfected with corresponding plasmid, after 48 h, adapted to suspension conditions and lysed in NETEN buffer (150 mmol/L NaCl, 20 mmol/L Tris–HCl (pH 7.40), 0.1% Nonidet P-40, 0.5 mmol/L EDTA, 1.5 mmol/L MgCl2, 10% glycerol) containing phospho-Stop, and protease inhibitor cocktail. The supernatants were incubated with anti-Flag-M2-Agarose beads (Sigma) or anti-HA beads (Sigma) for 2–4 h at 4℃. Besides, endogenous Co-IP was performed using MCF7 cells with anti-RNF8 antibodies or normal-mouse IgG, and the bound proteins were analyzed via immunoblotting. About the Ubiquitination assay, as the Co-IP described above, HEK293T cells were transfected with the corresponding plasmid and performed with Flag-IP, the elution was analyzed via western blotting, anti-Myc antibody, anti-YBX1 antibody, and RNF8 antibody were used to detect the Ubiquitin-YBX1, YBX1, and RNF8 protein expression level respectively.

#### Western blotting

Total proteins of cells were extracted using RIPA lysis buffer (Beyotime) with Protease Inhibitor (Roche) and Phosphatase Inhibitor (Roche). Protein samples were separated in sodium dodecyl sulfate (SDS)-PAGE and transferred to polyvinylidene fluoride (PVDF) filter membranes (Millipore, USA) for immune-hybridization. After 1 h of blocking in PBST (phosphate buffer saline containing 0.05% Tween-20 and 5% non-fat milk powder), the membranes were incubated with one of the following primary antibodies with corresponding concentration: RNF8 (Santa Cruz Biotech, 1:500), YBX1 (Cell Signaling Technology, 1:1000), HA (Convance, 1:1000), Secondary antibodies were Horseradish peroxidase (HRP)-conjugated anti-mouse IgG (ZB-2305, ZSGB-Bio, 1:4000) or anti-Rabbit IgG(Fc) (ZB-2301, ZSGB-Bio, 1:4000). Subsequently, band visualization was performed by electro-chemiluminescence (ECL) and detected by Digit imaging system (Thermo, Japan), the gray level of the bands was quantitated by ImageJ software.

### Statistical analysis

Statistical analysis was conducted using the GraphPad Prism. All results were presented as the mean ± standard error of the mean (SEM). *P* values are indicated in the text and figures above the two groups compared and *P* < 0.05 (denoted by asterisks) was considered as statistically significant.

## Supplementary Information

**Additional file 1. Figure S1.** The expression pattern of RNF8 in different cancers. (**A**) The expression profile of RNF8 across all tumor samples and normal tissues analyzed by TIMER database. (**B**) the comparsion of RNF8 in all datasets of Oncomine database. Numers represent the number of dataset. Red color represent significant overexpression in breast cancer tissues compared to normal tissues, blue represents low expression of RNF8. (**C**) the expression of RNF8 in all breast cancer datasets from Oncomine databse.

**Additional file 2. Figure S2.** The association between RNF8 expression and clinical parameters in breast cancer.

**Additional file 3. Figure S3.** The correlation between RNF8 expression and diseases.

**Additional file 4. Figure S4.** The PPI network using RNF8 and identified interactome.

**Additional file 5. Figure S5.** Functional PPI modules identified by Metascape.

**Additional file 6. Figure S6.** RNF8 abundance-based classification of cancer samples.

**Additional file 7. Figure S7.** Wikipathway analysis of RNF8 in cancers Group1.

**Additional file 8. Figure S8.** Functional analysis of RNF8 in cancers Group2.

**Additional file 9. Figure S9.** Upsetplot and Venn diagrame showing the intersection between five PPI database-generated RNF8-interacting proteins.

**Additional file 10. Figure S10. The survival analysis of hub proteins.** The heatmap of the pan-cancer OS rate of 11 hub proteins by Kaplan-Meier survival analysis based on TCGA samples by GEPIA. A log rank p <0.05 was considered to indicate a statistically significant difference and are framed in red (positively correlated) or blue (negatively correlated).

**Additional file 11. Supplementary information_1:** All supplementary figures and tables.

**Additional file 12. Supplementary information_2:** All targets identified by databases and LC-MS.

**Additional file 13. Supplementary information_3:** Proteomics data.

**Additional file 14. Supplementary information_4:** All original gel images.

**Additional file 15. Table S1.** 218 proteins identified by intersection of two dataset.

**Additional file 16. Table S2.** Hub genes list identified from online tools and LC-MS-based identification.

## Data Availability

All the data and materials supporting the conclusions are included in the main paper and additional materials.

## Abbreviations

GO: Gene Ontology
KEGG: Kyoto Encyclopedia of Genes and Genomes
TCGA: The Cancer Genome Atlas Project
GEO: Gene Expression Omnibus
LUAD: Lung adenocarcinoma
EMT: Epithelial–mesenchymal transition
STRING: The Search Tool for the Retrieval of Interacting Genes
PPI: Protein–protein interaction
GEPIA: Gene Expression Profiling Interactive Analysis
GTEx: Genotype-Tissue Expression
MFs: Molecular functions
BPs: Biological process
CCs: Cellular compounds

## References

[1] Nazari M, Babakhanzadeh E, Zarch SMA, Talebi M, Narimani N, Dargahi M (2020). Upregulation of the RNF8 gene can predict the presence of sperm in azoospermic individuals. Clin Exp Reprod Med.

[2] Kuang J, Min L, Liu C, Chen S, Gao C, Ma J (2020). RNF8 promotes epithelial–mesenchymal transition in lung cancer cells via stabilization of slug. Mol Cancer Res.

[3] Kuang J, Li L, Guo L, Su Y, Wang Y, Xu Y (2016). RNF8 promotes epithelial–mesenchymal transition of breast cancer cells. J Exp Clin Cancer Res.

[4] Lee H-J, Li C-F, Ruan D, Powers S, Thompson Patricia A, Frohman Michael A (2016). The DNA damage transducer RNF8 facilitates cancer chemoresistance and progression through twist activation. Mol Cell.

[5] Zhou T, Yi F, Wang Z, Guo Q, Liu J, Bai N (2019). The functions of DNA damage factor RNF8 in the pathogenesis and progression of cancer. Int J Biol Sci.

[6] Tsai Linda J, Lopezcolorado Felicia W, Bhargava R, Mendez-Dorantes C, Jahanshir E, Stark Jeremy M (2020). RNF8 has both KU-dependent and independent roles in chromosomal break repair. Nucleic Acids Res.

[7] Chen H, Shan J, Liu J, Feng Y, Ke Y, Qi W (2020). RNF8 promotes efficient DSB repair by inhibiting the pro-apoptotic activity of p53 through regulating the function of Tip60. Cell Prolifer.

[8] Nakajima NI, Yamauchi M, Kakoti S, Cuihua L, Kato R, Permata TBM (2020). RNF8 promotes high linear energy transfer carbon-ion-induced DNA double-stranded break repair in serum-starved human cells. DNA Repair.

[9] Baarends WM, Hoogerbrugge JW, Roest HP, Ooms M, Vreeburg J, Hoeijmakers JHJ (1999). Histone ubiquitination and chromatin remodeling in mouse spermatogenesis. Dev Biol.

[10] Gao S, Wu J, Liang L, Xu R (2017). RNF8 negatively regulates NF-kappaB signaling by targeting IkappaB kinase: implications for the regulation of inflammation signaling. Biochem Biophys Res Commun.

[11] Li L, Halaby M-J, Hakem A, Cardoso R, Ghamrasni SE, Harding S (2010). Rnf8 deficiency impairs class switch recombination, spermatogenesis, and genomic integrity and predisposes for cancer. J Exp Med.

[12] Rai R, Li J-M, Zheng H, Lok GT-M, Deng Y, Huen MSY (2011). The E3 ubiquitin ligase Rnf8 stabilizes Tpp1 to promote telomere end protection. Nat Struct Mol Biol.

[13] Lee C-C, Lin J-C, Hwang W-L, Kuo Y-J, Chen H-K, Tai S-K (2018). Macrophage-secreted interleukin-35 regulates cancer cell plasticity to facilitate metastatic colonization. Nat Commun.

[14] Du X, Zhang Z, Zheng X, Zhang H, Dong D, Zhang Z (2020). An electrochemical biosensor for the detection of epithelial–mesenchymal transition. Nat Commun.

[15] Su M, Xiao Y, Ma J, Tang Y, Tian B, Zhang Y (2019). Circular RNAs in Cancer: emerging functions in hallmarks, stemness, resistance and roles as potential biomarkers. Mol Cancer.

[16] Xu Y (2021). RNF8-mediated regulation of Akt promotes lung cancer cell survival and resistance to DNA damage. Cell reports.

[17] Zhao M-J, Song Y-F, Niu H-T, Tian Y-X, Yang X-G, Xie K (2016). Adenovirus-mediated downregulation of the ubiquitin ligase RNF8 sensitizes bladder cancer to radiotherapy. Oncotarget.

[18] Zhu Y, Qu C, Hong X, Jia Y, Lin M, Luo Y (2019). Trabid inhibits hepatocellular carcinoma growth and metastasis by cleaving RNF8-induced K63 ubiquitination of Twist1. Cell Death Differ.

[19] Liu C, Min L, Kuang J, Zhu C, Qiu X-Y, Zhu L (2019). Bioinformatic identification of miR-622 key target genes and experimental validation of the miR-622-RNF8 axis in breast cancer. Front Oncol.

[20] Min L, Liu C, Kuang J, Wu X, Zhu L (2019). miR-214 inhibits epithelial–mesenchymal transition of breast cancer cells via downregulation of RNF8. Acta Bioch Biophys Sin.

[21] Xu Y, Hu Y, Xu T, Yan K, Zhang T, Li Q (2021). RNF8-mediated regulation of Akt promotes lung cancer cell survival and resistance to DNA damage. Cell Rep.

[22] Zhou Y, Zhou B, Pache L, Chang M, Khodabakhshi AH, Tanaseichuk O (2019). Metascape provides a biologist-oriented resource for the analysis of systems-level datasets. Nat Commun.

[23] Fritsch J, Stephan M, Tchikov V, Winoto-Morbach S, Gubkina S, Kabelitz D (2014). Cell fate decisions regulated by K63 ubiquitination of tumor necrosis factor receptor 1. Mol Cell Biol.

[24] Hartley AV, Wang B, Mundade R, Jiang G, Sun M, Wei H (2020). PRMT5-mediated methylation of YBX1 regulates NF-κB activity in colorectal cancer. Sci Rep.

[25] Shibata T, Tokunaga E, Hattori S, Watari K, Murakami Y, Yamashita N (2018). Y-box binding protein YBX1 and its correlated genes as biomarkers for poor outcomes in patients with breast cancer. Oncotarget.

[26] Feng L, Chen J (2012). The E3 ligase RNF8 regulates KU80 removal and NHEJ repair. Nat Struct Mol Biol.

[27] Pickart CM (2001). Mechanisms underlying ubiquitination. Annu Rev Biochem.

[28] Zhou H, Mu X, Chen J, Liu H, Shi W, Xing E (2013). RNAi silencing targeting RNF8 enhances radiosensitivity of a non-small cell lung cancer cell line A549. Int J Radiat Biol.

[29] Valnegri P, Huang J, Yamada T, Yang Y, Mejia LA, Cho HY (2017). RNF8/UBC13 ubiquitin signaling suppresses synapse formation in the mammalian brain. Nat Commun.

[30] Suresh PS, Tsutsumi R, Venkatesh T (2018). YBX1 at the crossroads of non-coding transcriptome, exosomal, and cytoplasmic granular signaling. Eur J Cell Biol.

[31] Wei WJ, Mu SR, Heiner M, Fu X, Cao LJ, Gong XF (2012). YB-1 binds to CAUC motifs and stimulates exon inclusion by enhancing the recruitment of U2AF to weak polypyrimidine tracts. Nucleic Acids Res.

[32] Gandhi M, Groß M, Holler JM, Coggins SAA, Patil N, Leupold JH (2020). The lncRNA lincNMR regulates nucleotide metabolism via a YBX1–RRM2 axis in cancer. Nat Commun.

[33] Goodarzi H, Liu X, Nguyen Hoang CB, Zhang S, Fish L, Tavazoie SF (2015). Endogenous tRNA-derived fragments suppress breast cancer progression via YBX1 displacement. Cell.

[34] Sin HS, Barski A, Zhang F, Kartashov AV, Nussenzweig A, Chen J (2012). RNF8 regulates active epigenetic modifications and escape gene activation from inactive sex chromosomes in post-meiotic spermatids. Genes Dev.

[35] Sanna S, Esposito S, Masala A, Sini P, Nieddu G, Galioto M (2020). HDAC1 inhibition ameliorates TDP-43-induced cell death in vitro and in vivo. Cell Death Dis.

[36] Ouyang S, Song Y, Tian Y, Chen Y, Yu X, Wang D (2015). RNF8 deficiency results in neurodegeneration in mice. Neurobiol Aging.

[37] Kolas NK, Chapman JR, Nakada S, Ylanko J, Chahwan R, Sweeney FD (2007). Orchestration of the DNA-damage response by the RNF8 ubiquitin ligase. Science.

[38] Huen MSY, Grant R, Manke I, Minn K, Yu X, Yaffe MB (2007). RNF8 Transduces the DNA-damage signal via histone ubiquitylation and checkpoint protein assembly. Cell.

[39] Watanabe S, Watanabe K, Akimov V, Bartkova J, Blagoev B, Lukas J (2013). JMJD1C demethylates MDC1 to regulate the RNF8 and BRCA1-mediated chromatin response to DNA breaks. Nat Struct Mol Biol.

[40] Chen H, Shan J, Liu J, Feng Y, Zeng XJCP (2020). RNF8 promotes efficient DSB repair by inhibiting the pro-apoptotic activity of p53 through regulating the function of Tip60. Cell Prolif.

[41] Mandemaker IK, van Cuijk L, Janssens RC, Lans H, Bezstarosti K, Hoeijmakers JH (2017). DNA damage-induced histone H1 ubiquitylation is mediated by HUWE1 and stimulates the RNF8-RNF168 pathway. Sci Rep.

[42] Wang S, Luo H, Wang C, Sun H, Sun G, Sun N (2017). RNF8 identified as a co-activator of estrogen receptor α promotes cell growth in breast cancer. Biochim Biophys Acta BBA Mol Basis Dis.

[43] Koch A, Meyer TD, Jeschke J, Criekinge WV (2015). MEXPRESS: visualizing expression, DNA methylation and clinical TCGA data. BMC Genomics.

[44] Koch A, Jeschke J, Van Criekinge W, van Engeland M, De Meyer T (2019). MEXPRESS update 2019. Nucleic Acids Res.

[45] Koch A, De Meyer T, Jeschke J, Van Criekinge W (2015). MEXPRESS: visualizing expression, DNA methylation and clinical TCGA data. BMC Genomics.

[46] Rhodes DR, Yu J, Shanker K, Deshpande N, Varambally R, Ghosh D (2004). ONCOMINE: a cancer microarray database and integrated data-mining platform. Neoplasia.

[47] Warde-Farley D, Donaldson SL, Comes O, Zuberi K, Badrawi R, Chao P (2010). The GeneMANIA prediction server: biological network integration for gene prioritization and predicting gene function. Nucleic Acids Res.

[48] Damian S, Gable AL, David L, Alexander J, Stefan W, Jaime HC (2018). STRING v11: protein–protein association networks with increased coverage, supporting functional discovery in genome-wide experimental datasets. Nucleic Acids Res.

[49] López Y, Nakai K, Patil A (2015). HitPredict version 4: comprehensive reliability scoring of physical protein–protein interactions from more than 100 species. Database (Oxford).

[50] Stark C, Breitkreutz BJ, Reguly T, Boucher L, Breitkreutz A, Tyers M (2006). BioGRID: a general repository for interaction datasets. Nucleic Acids Res.

[51] Li T, Wernersson R, Hansen RB, Horn H, Mercer J, Slodkowicz G (2017). A scored human protein–protein interaction network to catalyze genomic interpretation. Nat Methods.

[52] Tang Z, Kang B, Li C, Chen T, Zhang Z (2019). GEPIA2: an enhanced web server for large-scale expression profiling and interactive analysis. Nucleic Acids Res.

[53] Xie ZC, Li TT, Gan BL, Gao X, Gao L, Chen G (2018). Investigation of miR-136-5p key target genes and pathways in lung squamous cell cancer based on TCGA database and bioinformatics analysis. Pathol Res Pract.

[54] Wang S, Xiong Y, Zhao L, Gu K, Li Y, Zhao F (2021). UCSCXenaShiny: an R/CRAN package for interactive analysis of UCSC xena data. Bioinformatics (Oxford, England).

