# Supplementary figures and images for: A functional reference map of the RNF8 interactome in cancer
Source: Biol Direct. 2022 Jul 13;17:17. doi: 10.1186/s13062-022-00331-z (PMC9277853; doi:10.1186/s13062-022-00331-z)

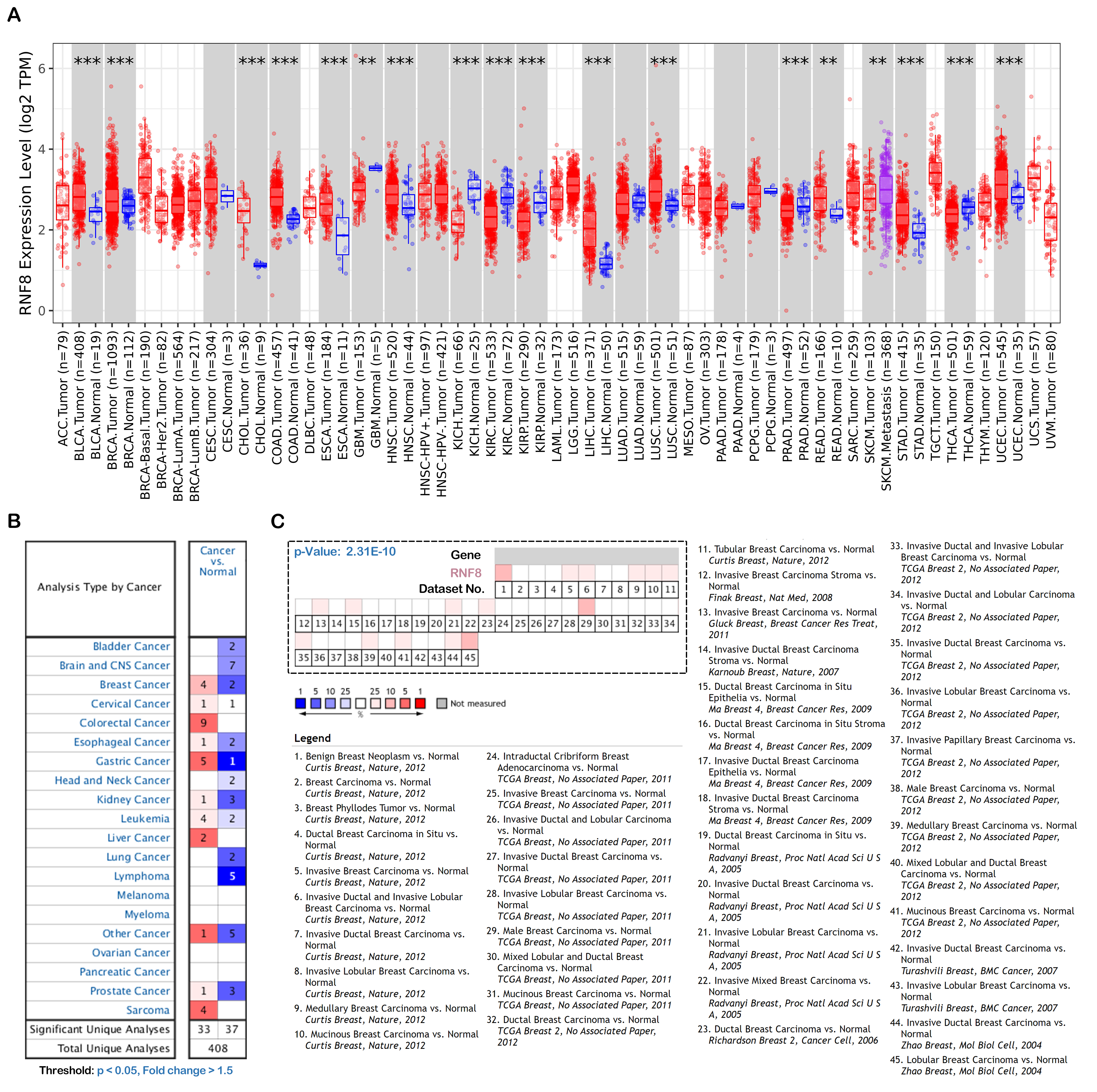

Supplement: Supplementary file 1 — Additional file 1. Figure S1. The expression pattern of RNF8 in different cancers. (A) The expression profile of RNF8 across all tumor samples and normal tissues analyzed by TIMER database. (B) the comparsion of RNF8 in all datasets of Oncomine database. Numers represent the number of dataset. Red color represent significant overexpression in breast cancer tissues compared to normal tissues, blue represents low expression of RNF8. (C) the expression of RNF8 in all breast cancer datasets from Oncomine databse. [file 13062_2022_331_MOESM1_ESM.png]

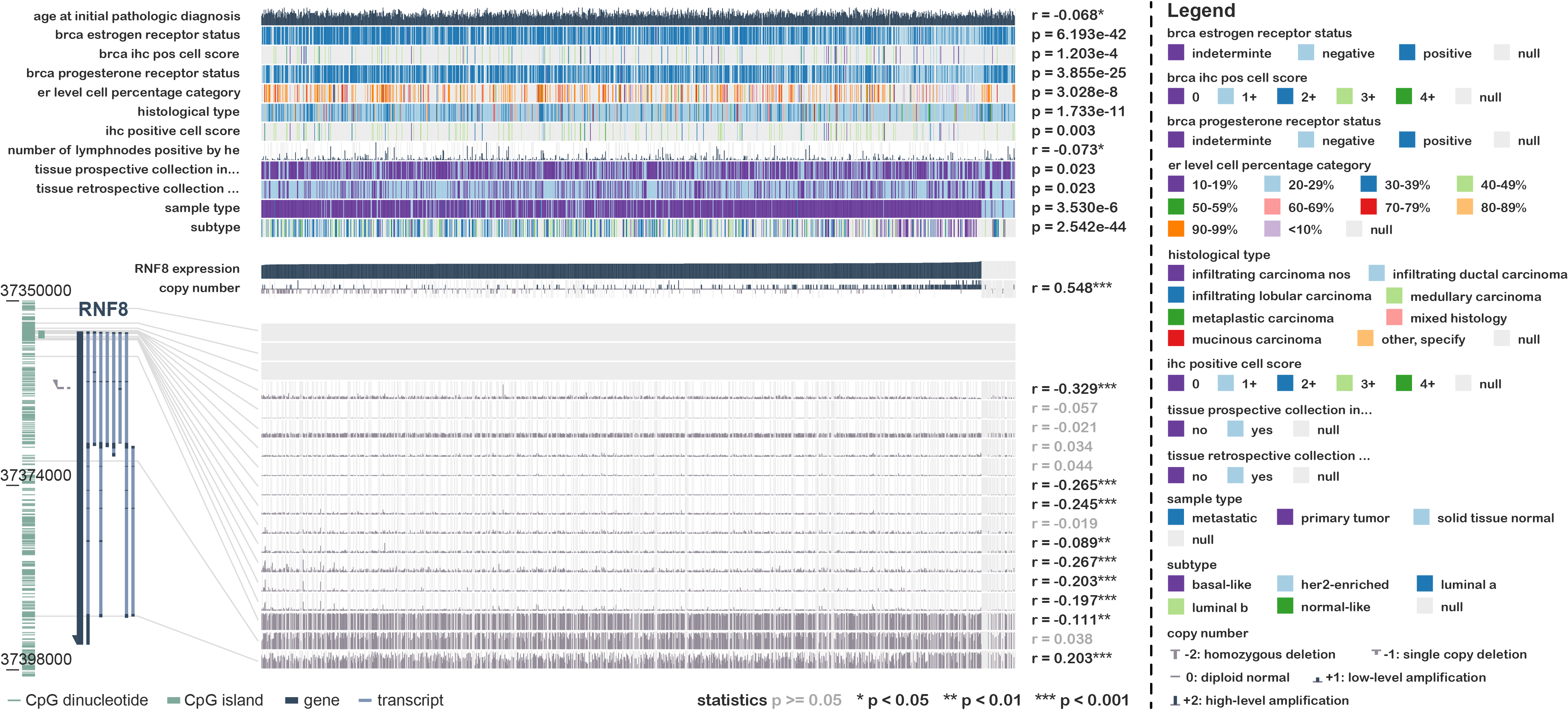

Supplement: Supplementary file 2 — Additional file 2. Figure S2. The association between RNF8 expression and clinical parameters in breast cancer. [file 13062_2022_331_MOESM2_ESM.png]

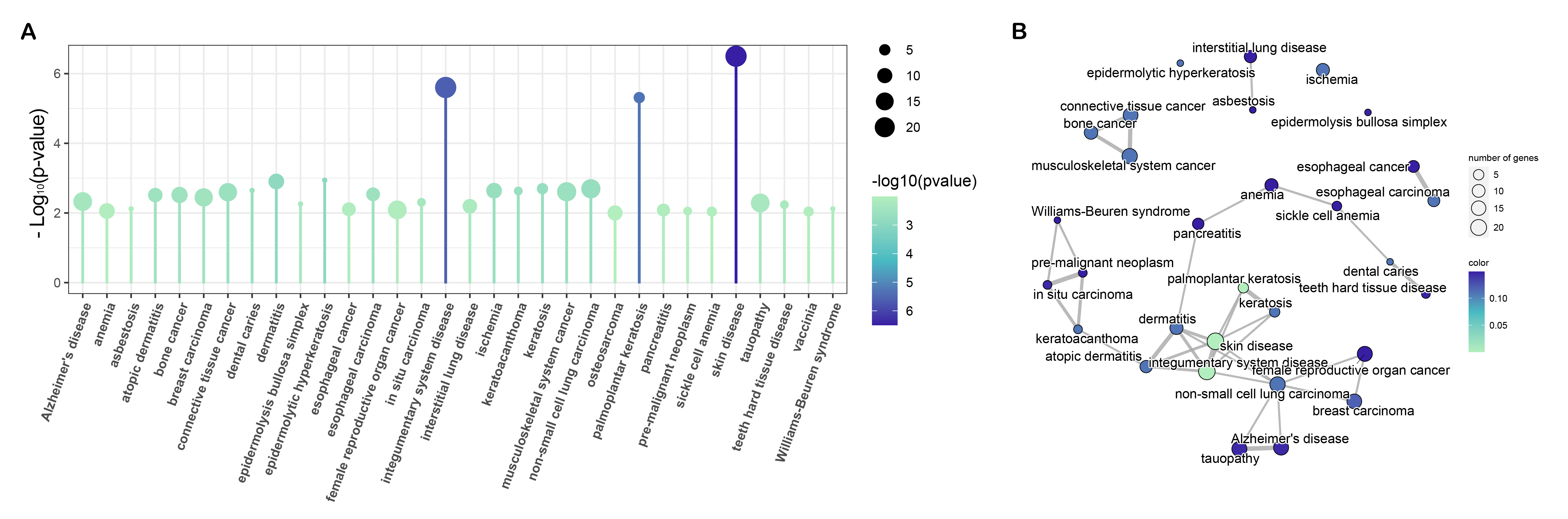

Supplement: Supplementary file 3 — Additional file 3. Figure S3. The correlation between RNF8 expression and diseases. [file 13062_2022_331_MOESM3_ESM.png]

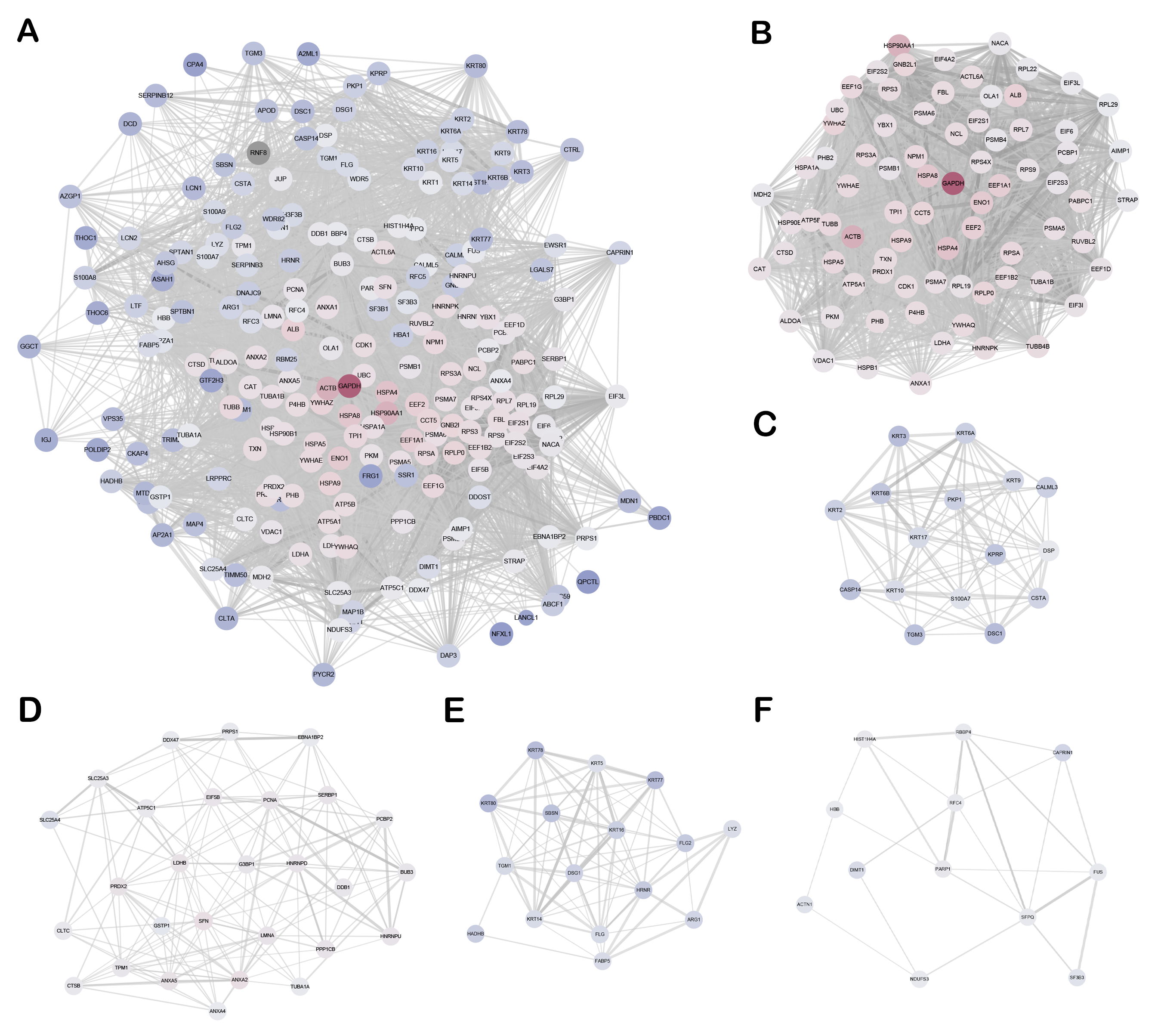

Supplement: Supplementary file 4 — Additional file 4. Figure S4. The PPI network using RNF8 and identified interactome. [file 13062_2022_331_MOESM4_ESM.png]

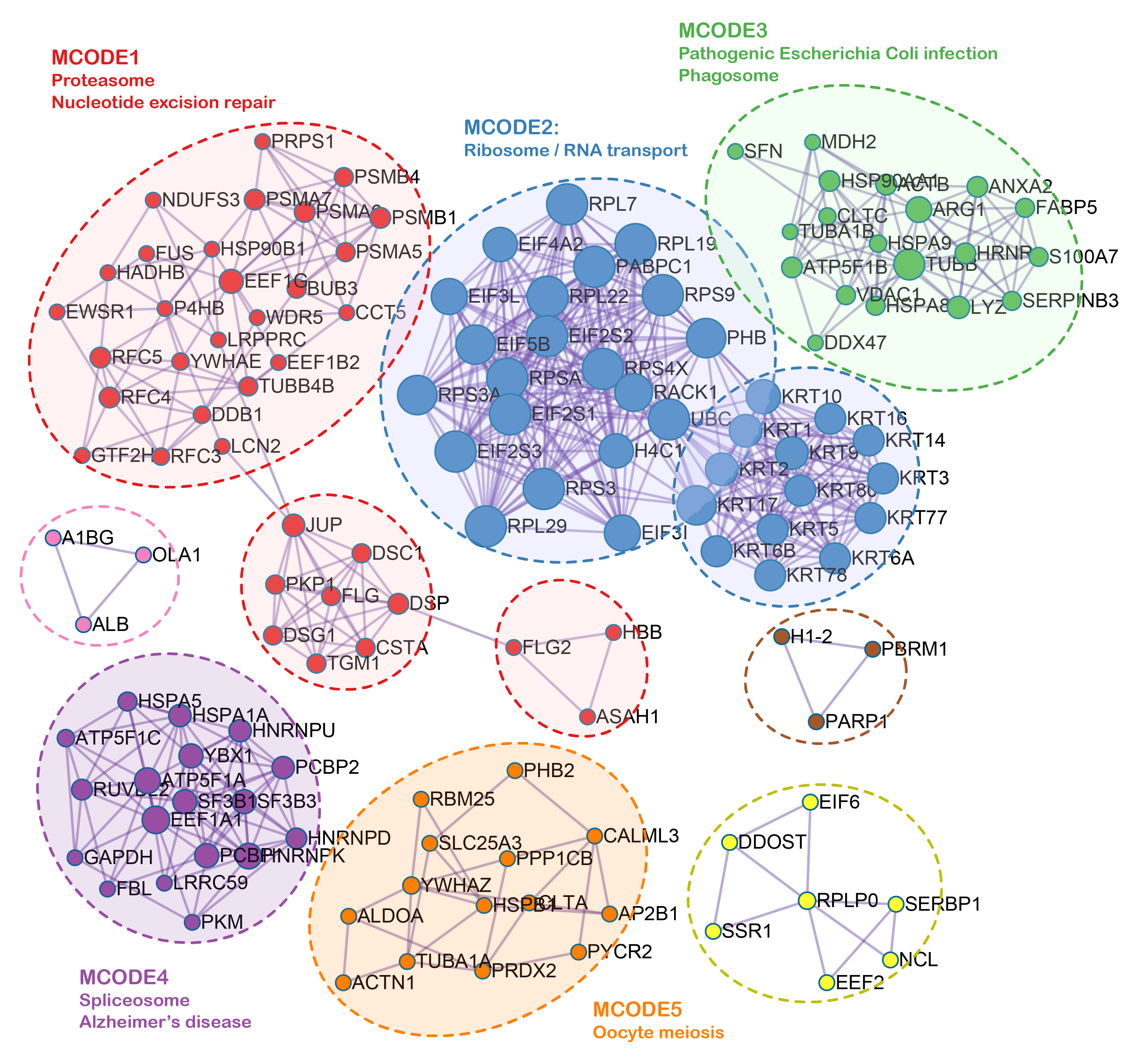

Supplement: Supplementary file 5 — Additional file 5. Figure S5. Functional PPI modules identified by Metascape. [file 13062_2022_331_MOESM5_ESM.png]

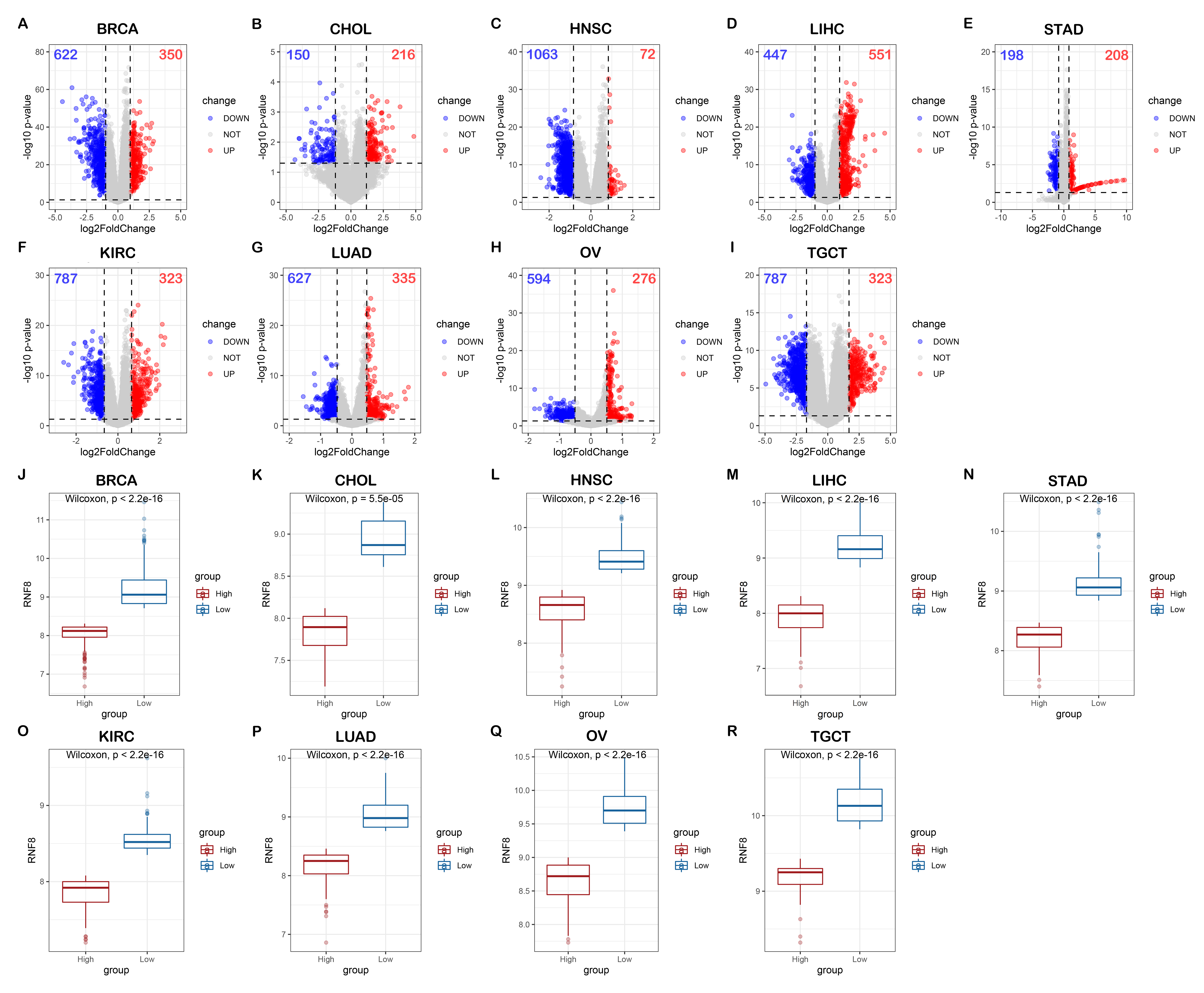

Supplement: Supplementary file 6 — Additional file 6. Figure S6. RNF8 abundance-based classification of cancer samples. [file 13062_2022_331_MOESM6_ESM.png]

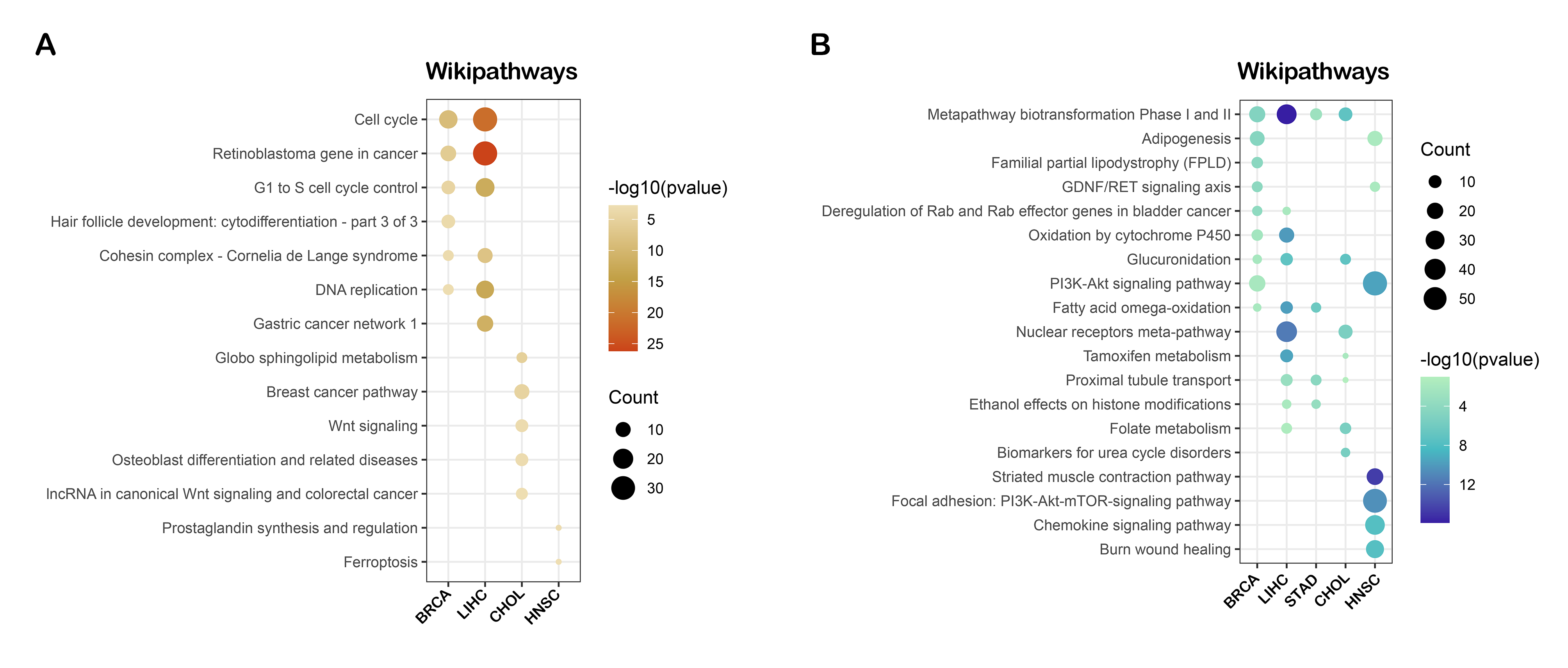

Supplement: Supplementary file 7 — Additional file 7. Figure S7. Wikipathway analysis of RNF8 in cancers Group1. [file 13062_2022_331_MOESM7_ESM.png]

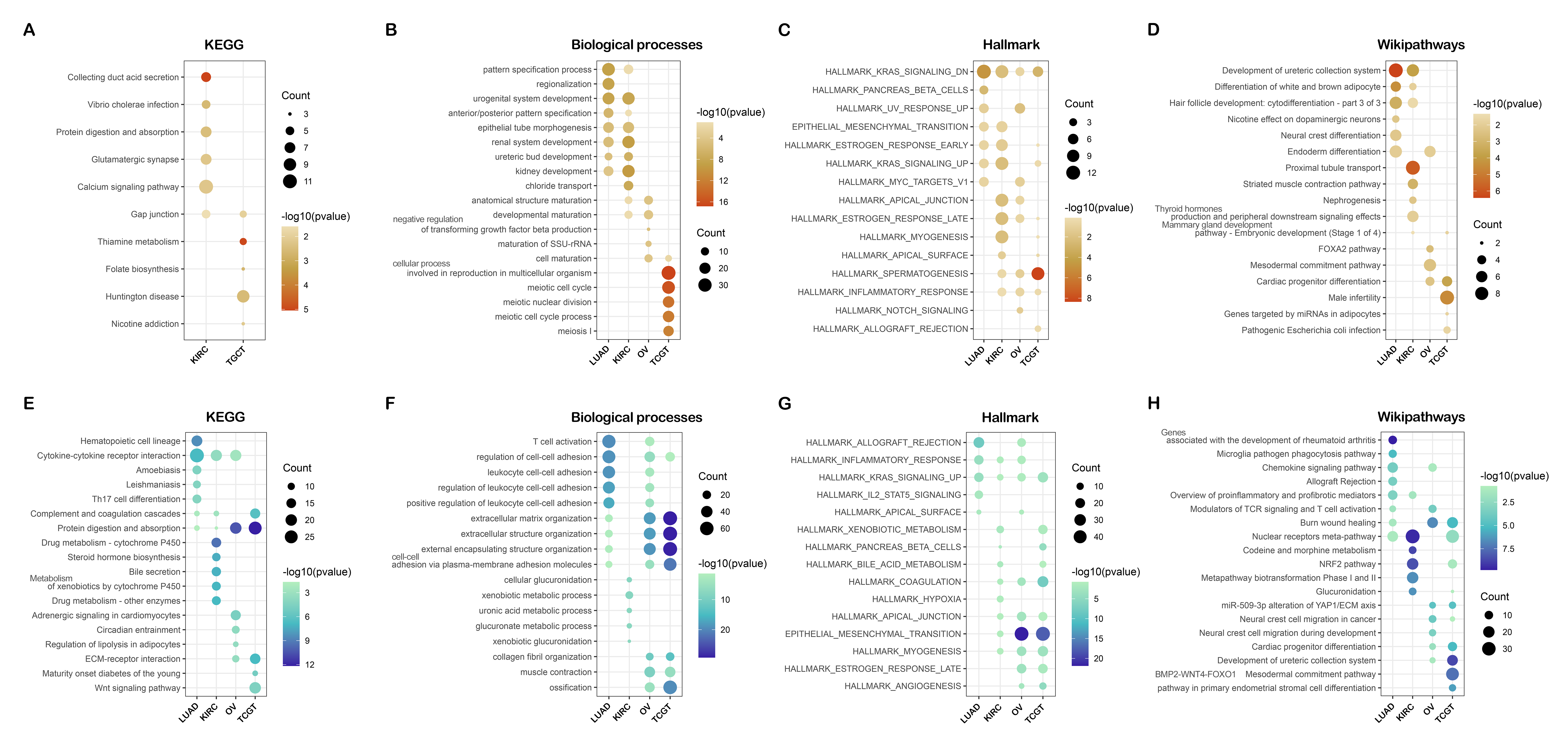

Supplement: Supplementary file 8 — Additional file 8. Figure S8. Functional analysis of RNF8 in cancers Group2. [file 13062_2022_331_MOESM8_ESM.png]

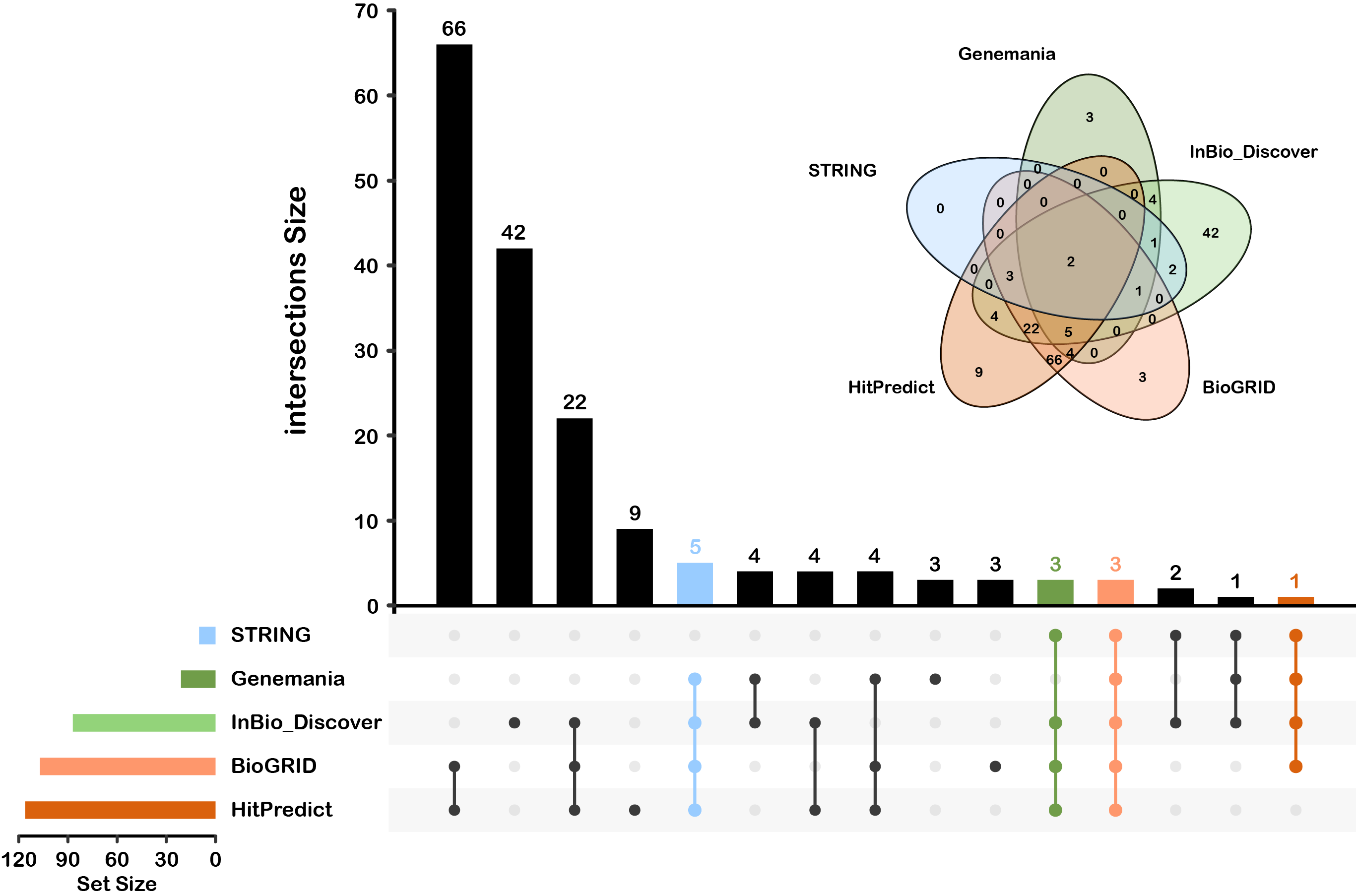

Supplement: Supplementary file 9 — Additional file 9. Figure S9. Upsetplot and Venn diagrame showing the intersection between five PPI database-generated RNF8-interacting proteins. [file 13062_2022_331_MOESM9_ESM.png]

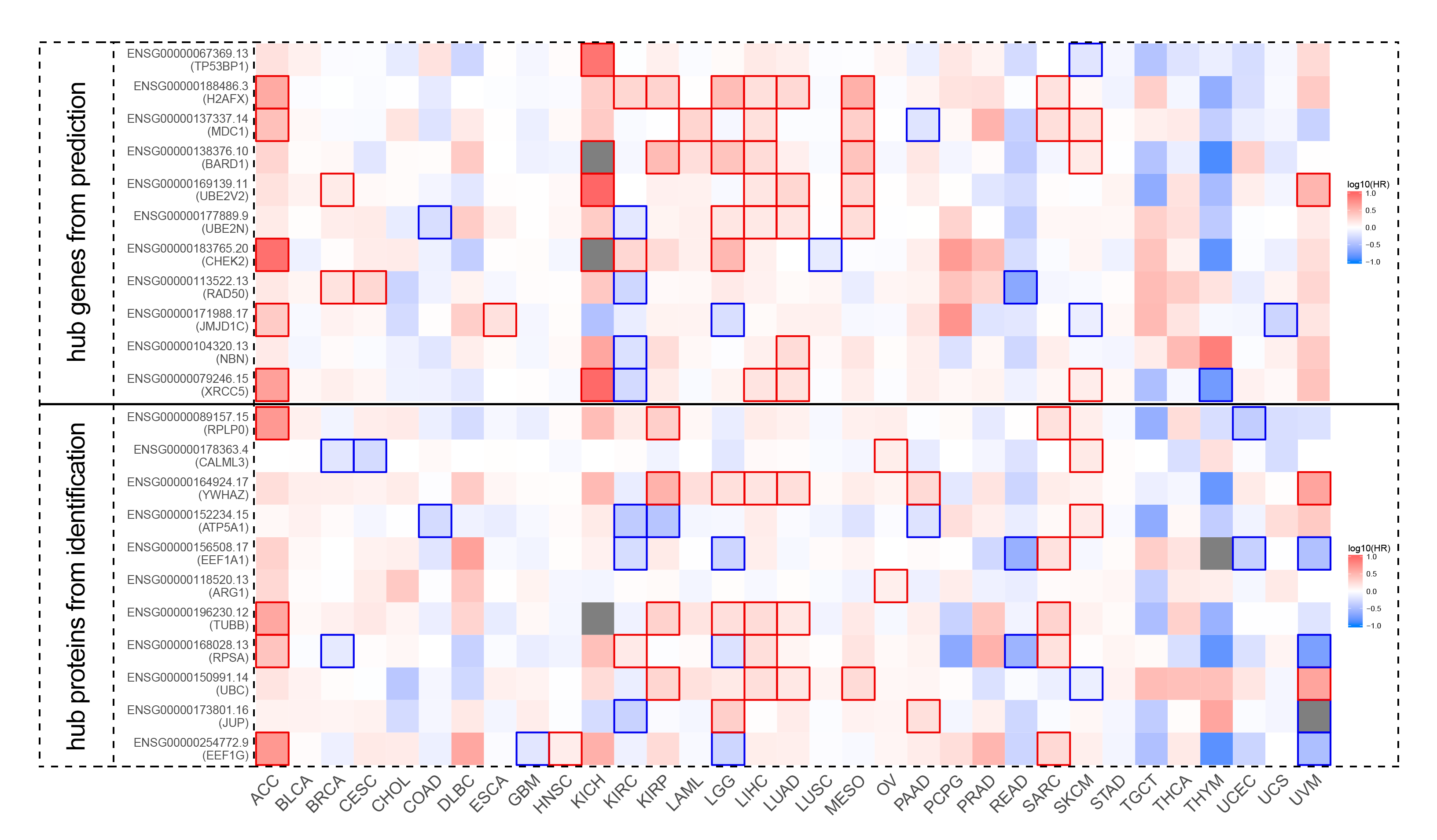

Supplement: Supplementary file 10 — Additional file 10. Figure S10. The survival analysis of hub proteins. The heatmap of the pan-cancer OS rate of 11 hub proteins by Kaplan-Meier survival analysis based on TCGA samples by GEPIA. A log rank p <0.05 was considered to indicate a statistically significant difference and are framed in red (positively correlated) or blue (negatively correlated). [file 13062_2022_331_MOESM10_ESM.png]

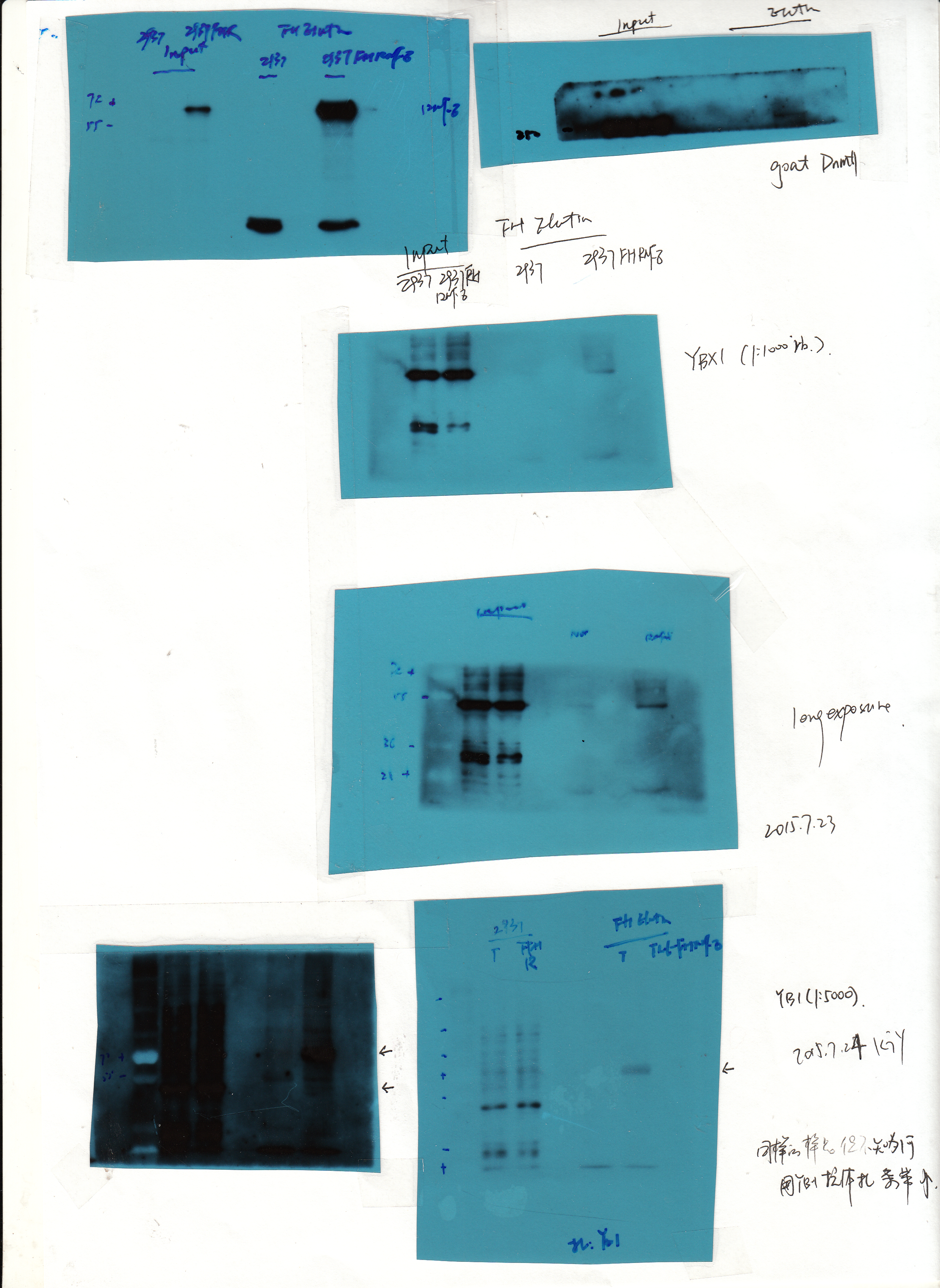

Supplement: Supplementary file 14 — Additional file 14. Supplementary information_4: All original gel images. [file 13062_2022_331_MOESM14_ESM.zip › TO CHECK/2015-07-15_0001.tif]

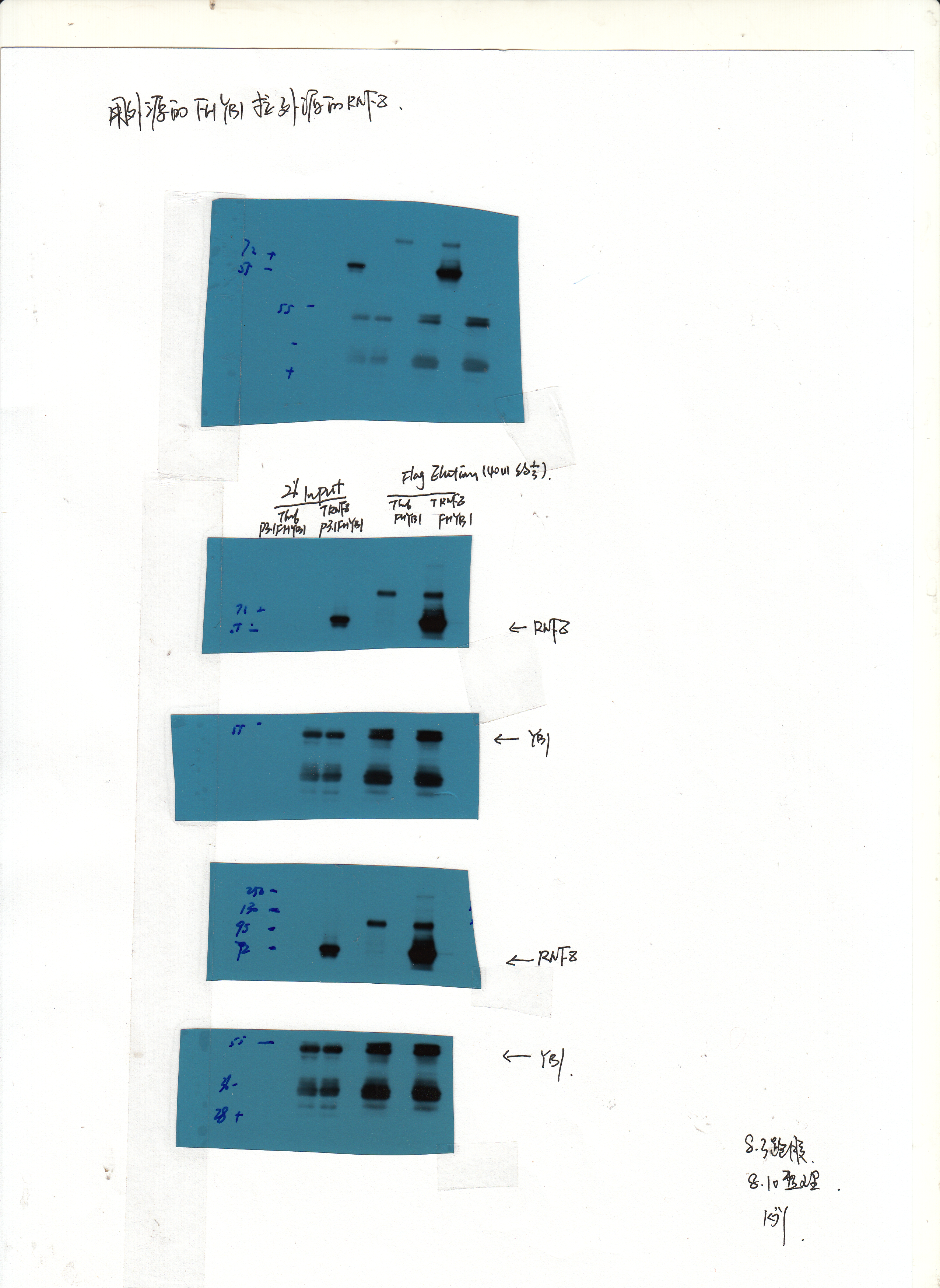

Supplement: Supplementary file 14 — Additional file 14. Supplementary information_4: All original gel images. [file 13062_2022_331_MOESM14_ESM.zip › TO CHECK/2015-07-15_0002.tif]

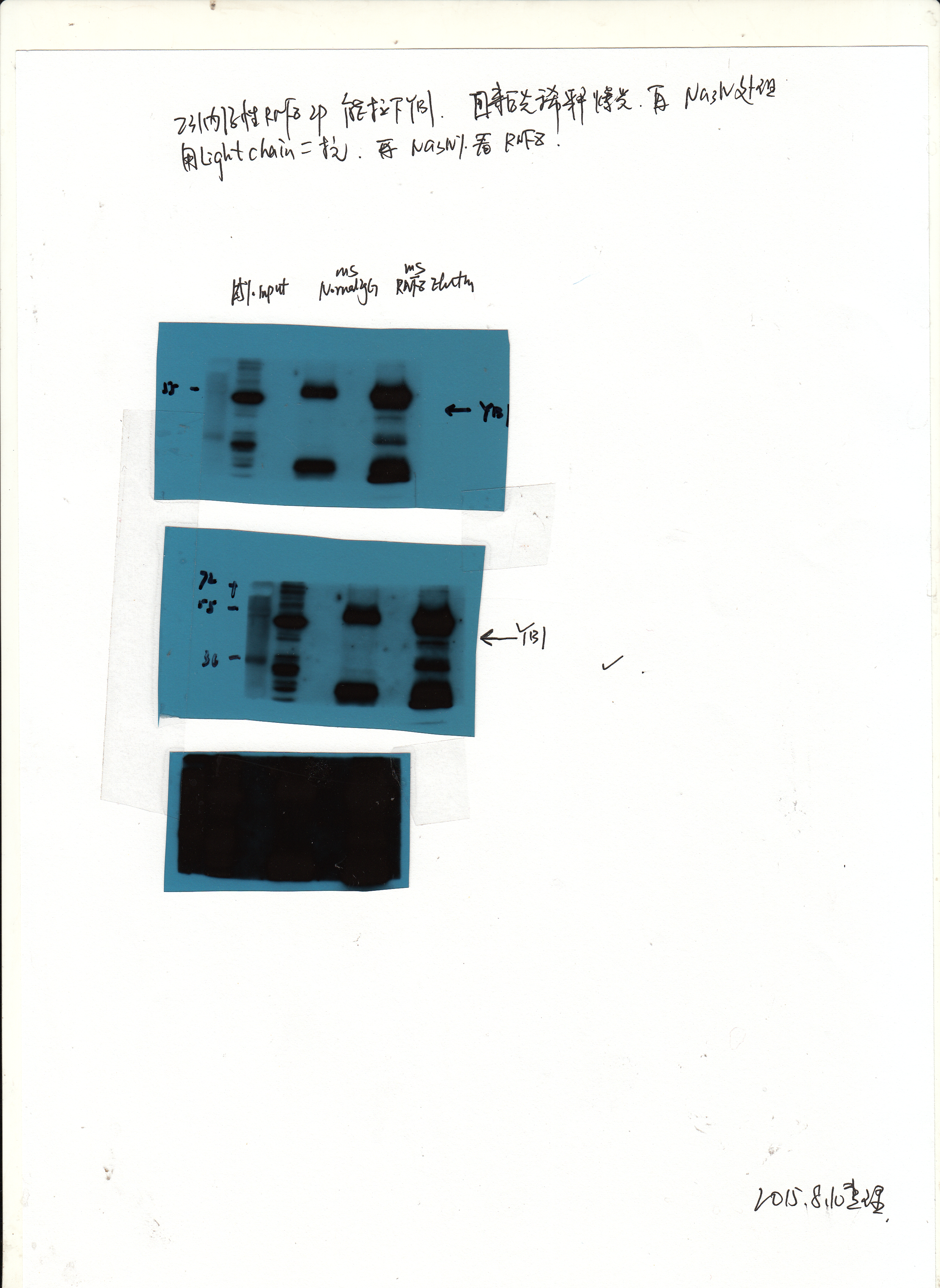

Supplement: Supplementary file 14 — Additional file 14. Supplementary information_4: All original gel images. [file 13062_2022_331_MOESM14_ESM.zip › TO CHECK/2015-07-15_0004.tif]

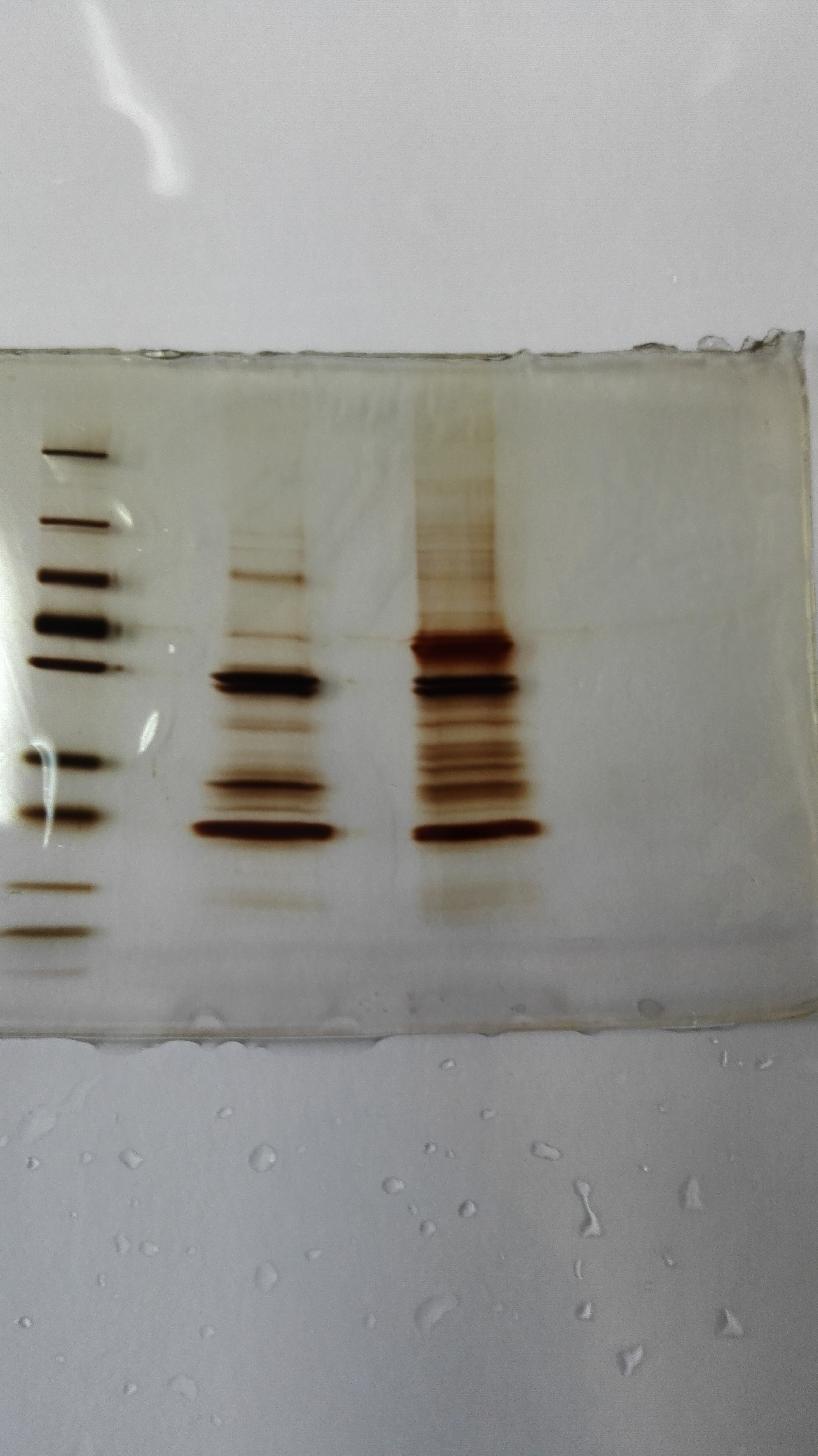

Supplement: Supplementary file 14 — Additional file 14. Supplementary information_4: All original gel images. [file 13062_2022_331_MOESM14_ESM.zip › TO CHECK/IMG_20150419_152041.jpg]

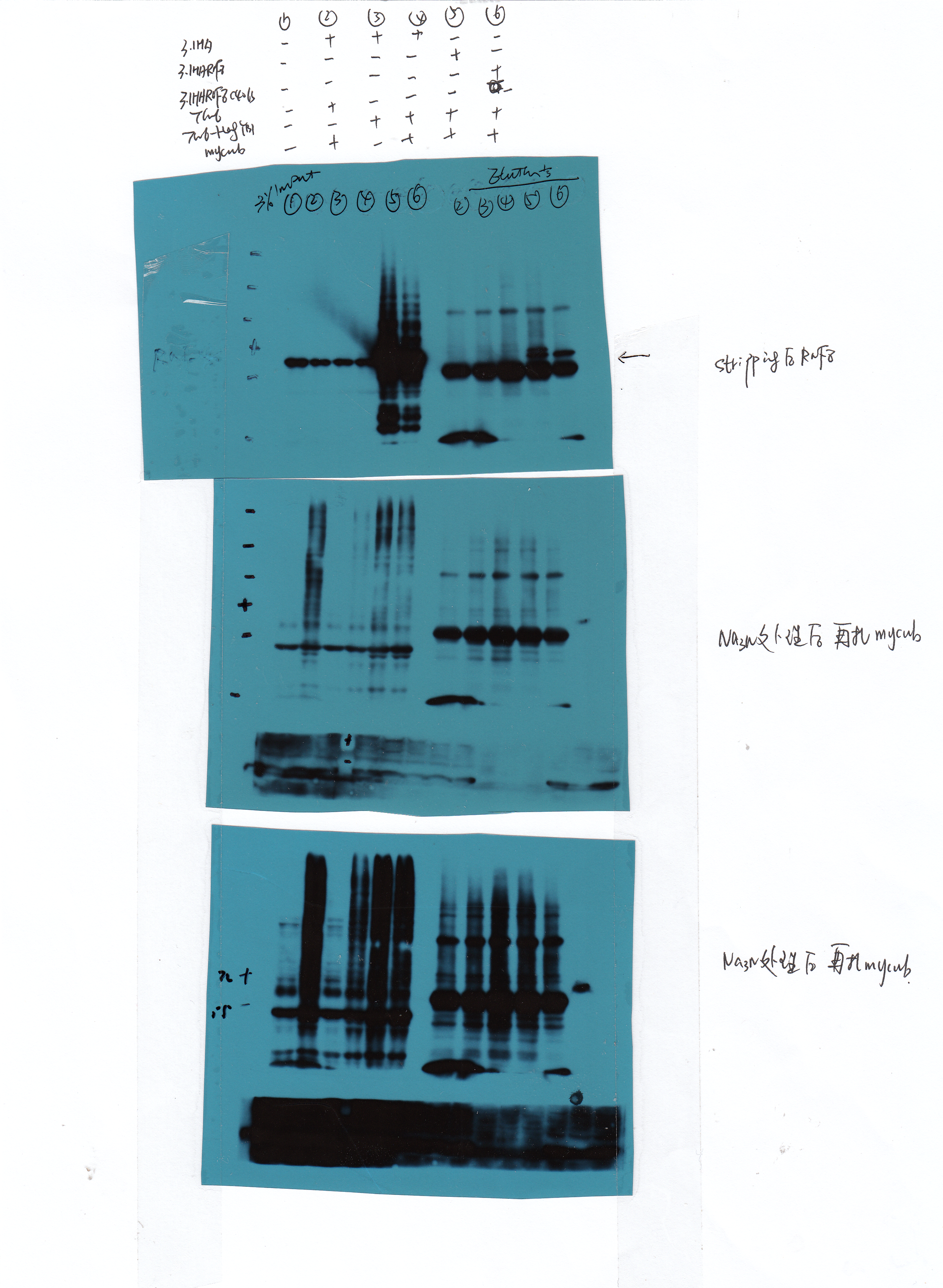

Supplement: Supplementary file 14 — Additional file 14. Supplementary information_4: All original gel images. [file 13062_2022_331_MOESM14_ESM.zip › TO CHECK/ubyb1.tif]
