# Supplementary material for: A functional reference map of the RNF8 interactome in cancer
Source: Biol Direct. 2022 Jul 13;17:17. doi: 10.1186/s13062-022-00331-z (PMC9277853; doi:10.1186/s13062-022-00331-z)
Supplement: Supplementary file 11 — Additional file 11. Supplementary information_1: All supplementary figures and tables. [file 13062_2022_331_MOESM11_ESM.docx]

**
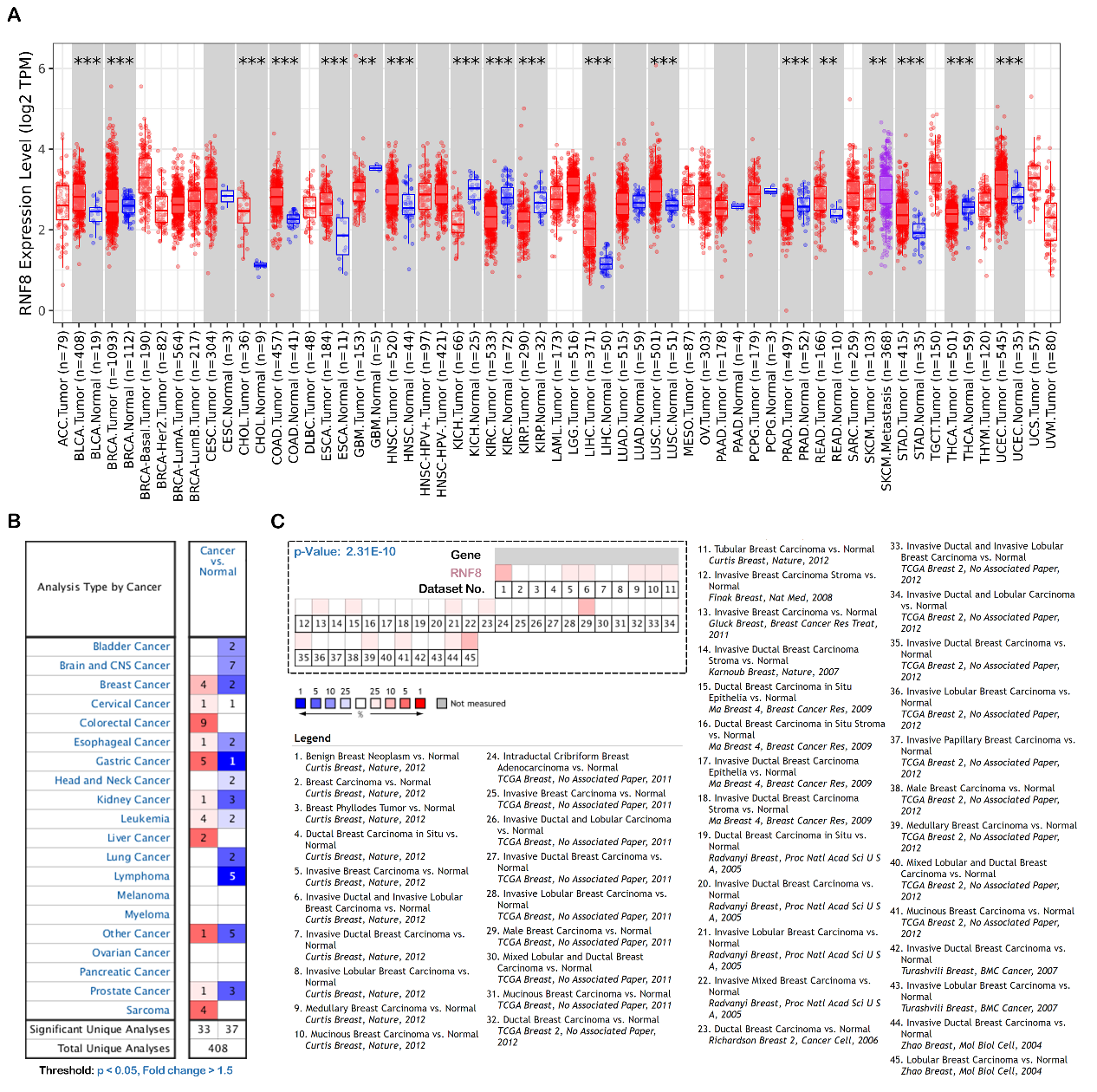
Supplementary informations**

**Figure S1. The expression pattern of RNF8 in different cancers.** (A) The expression profile of RNF8 across all tumor samples and normal tissues analyzed by TIMER database. (B) the comparsion of RNF8 in all datasets of Oncomine database. Numers represent the number of dataset. Red color represent significant overexpression in breast cancer tissues compared to normal tissues, blue represents low expression of RNF8. (C) the expression of RNF8 in all breast cancer datasets from Oncomine databse.


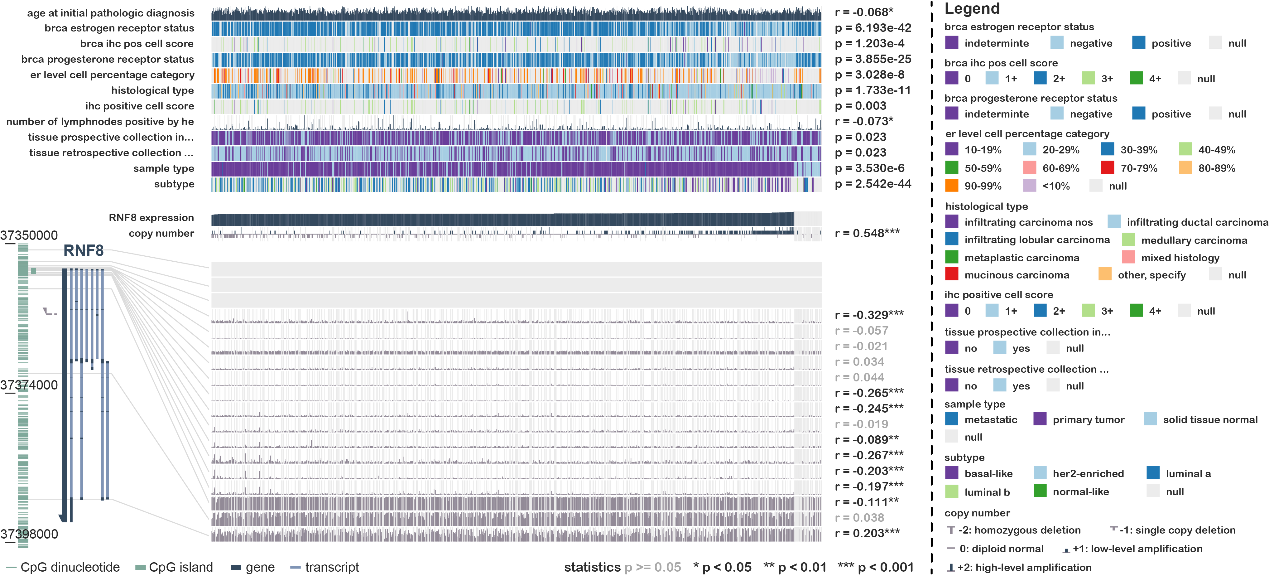


**Figure S2. The association between RNF8 expression and clinical parameters in breast cancer.**

**
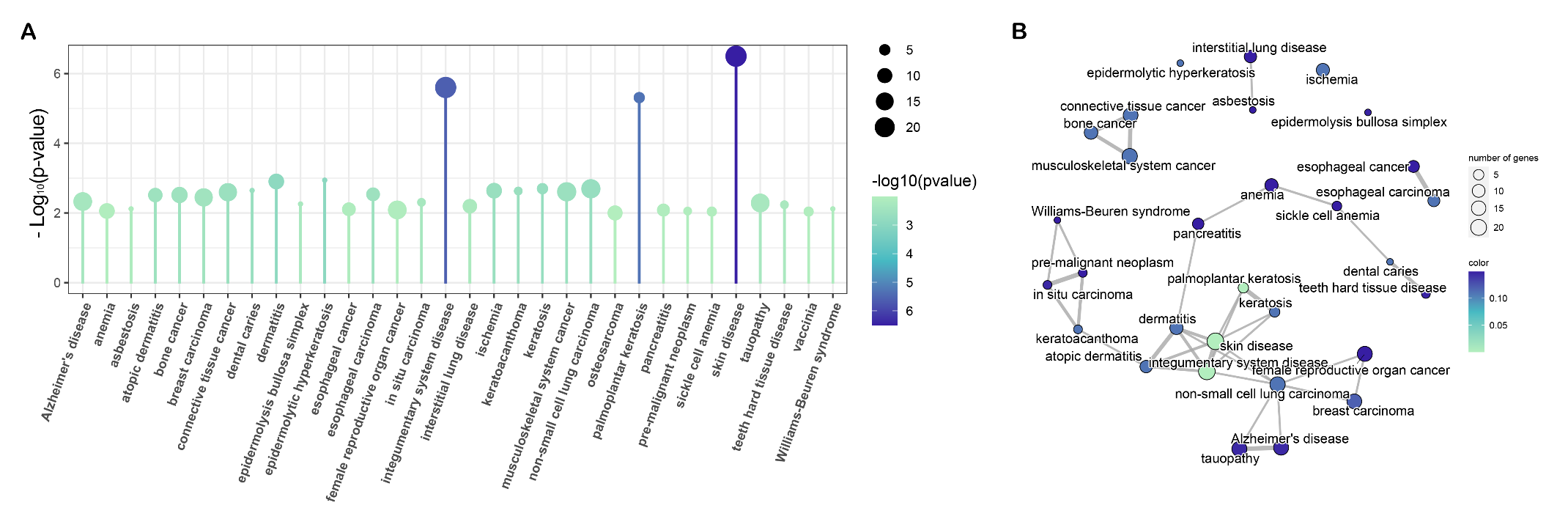
**

**Figure S3. The correlation between RNF8 expression and diseases.**

**
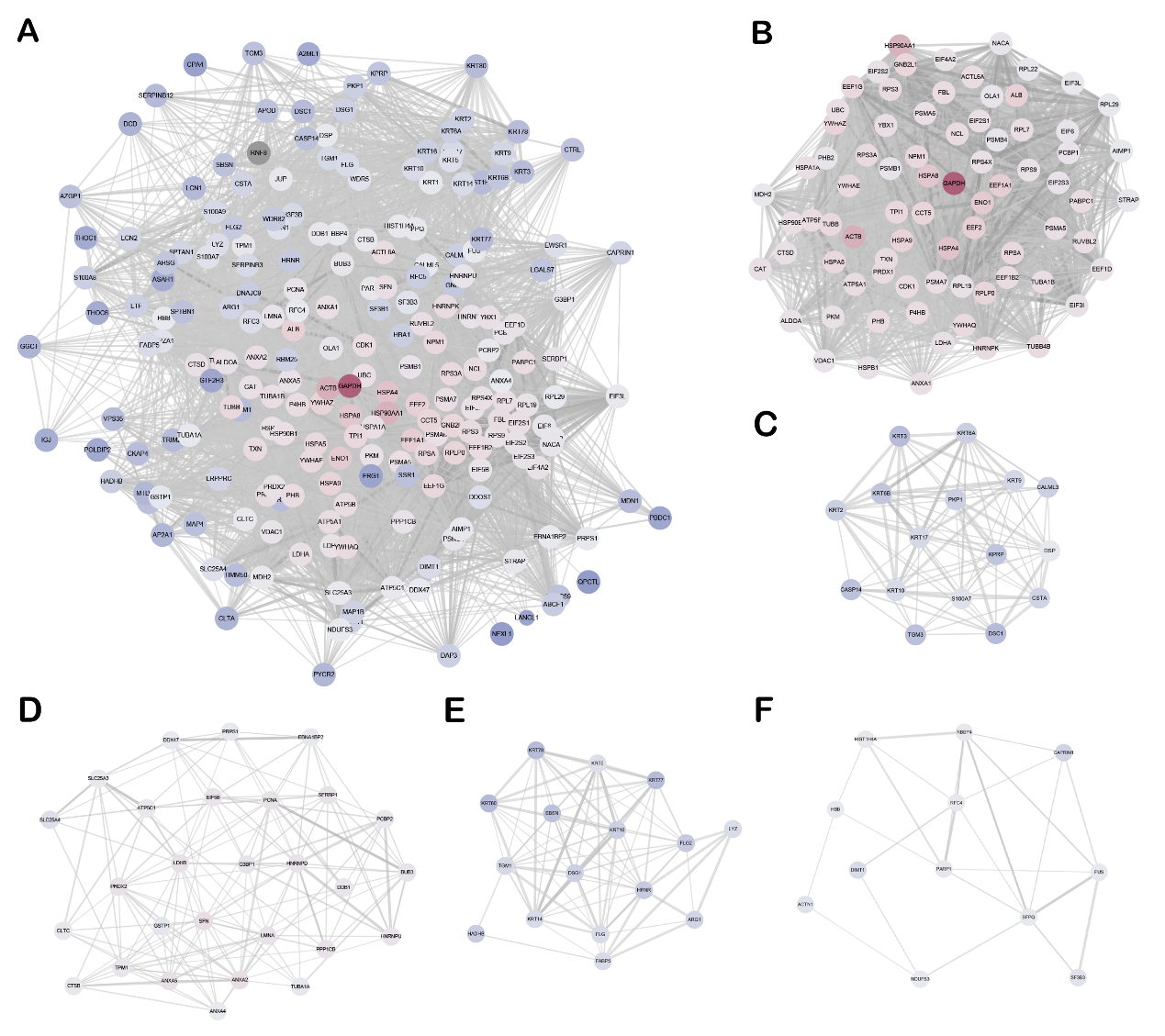
**

**Figure S4. The PPI network using RNF8 and identified interactome.**

**
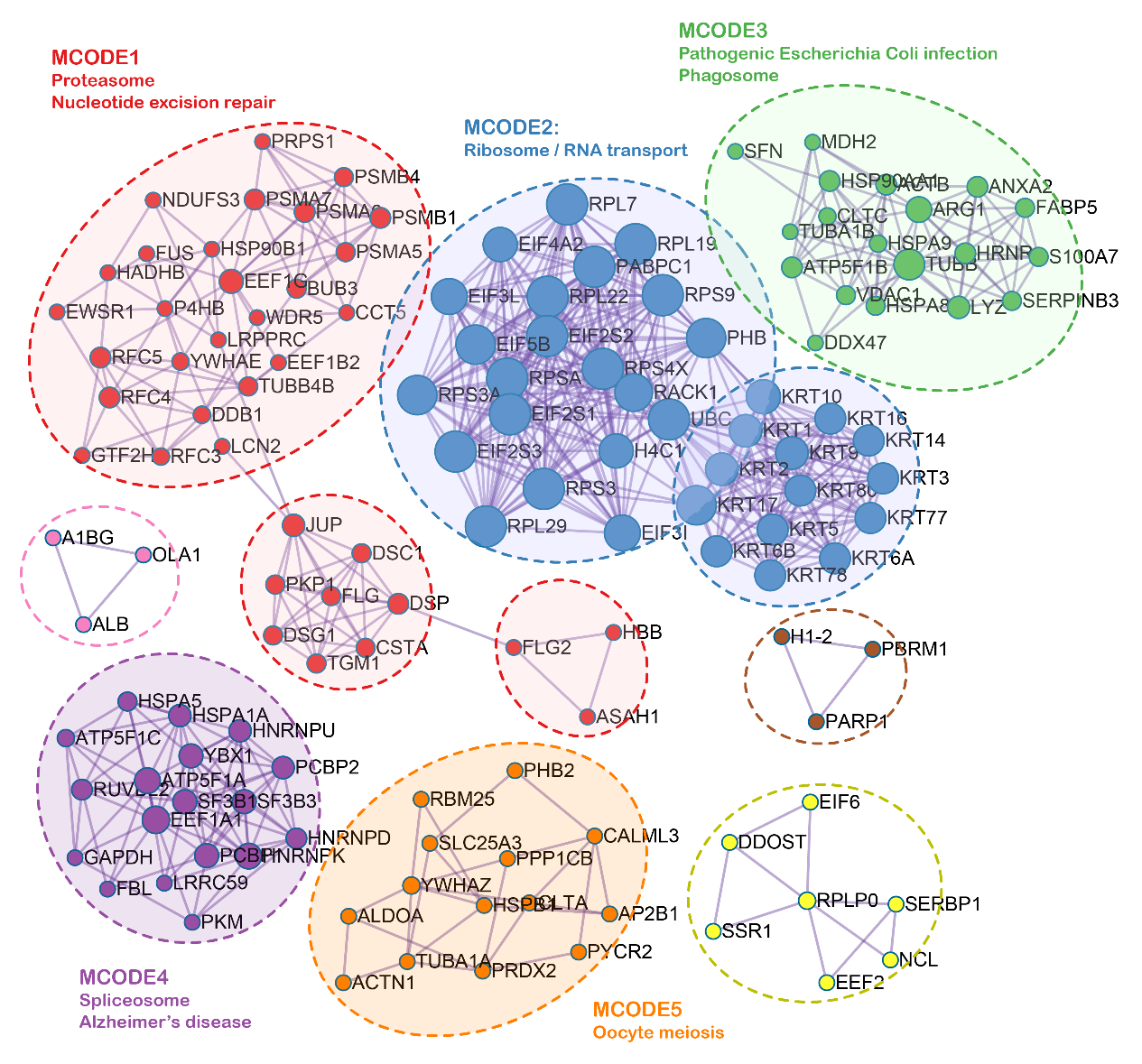
Figure S5. Functional PPI modules identified by Metascape.**

**
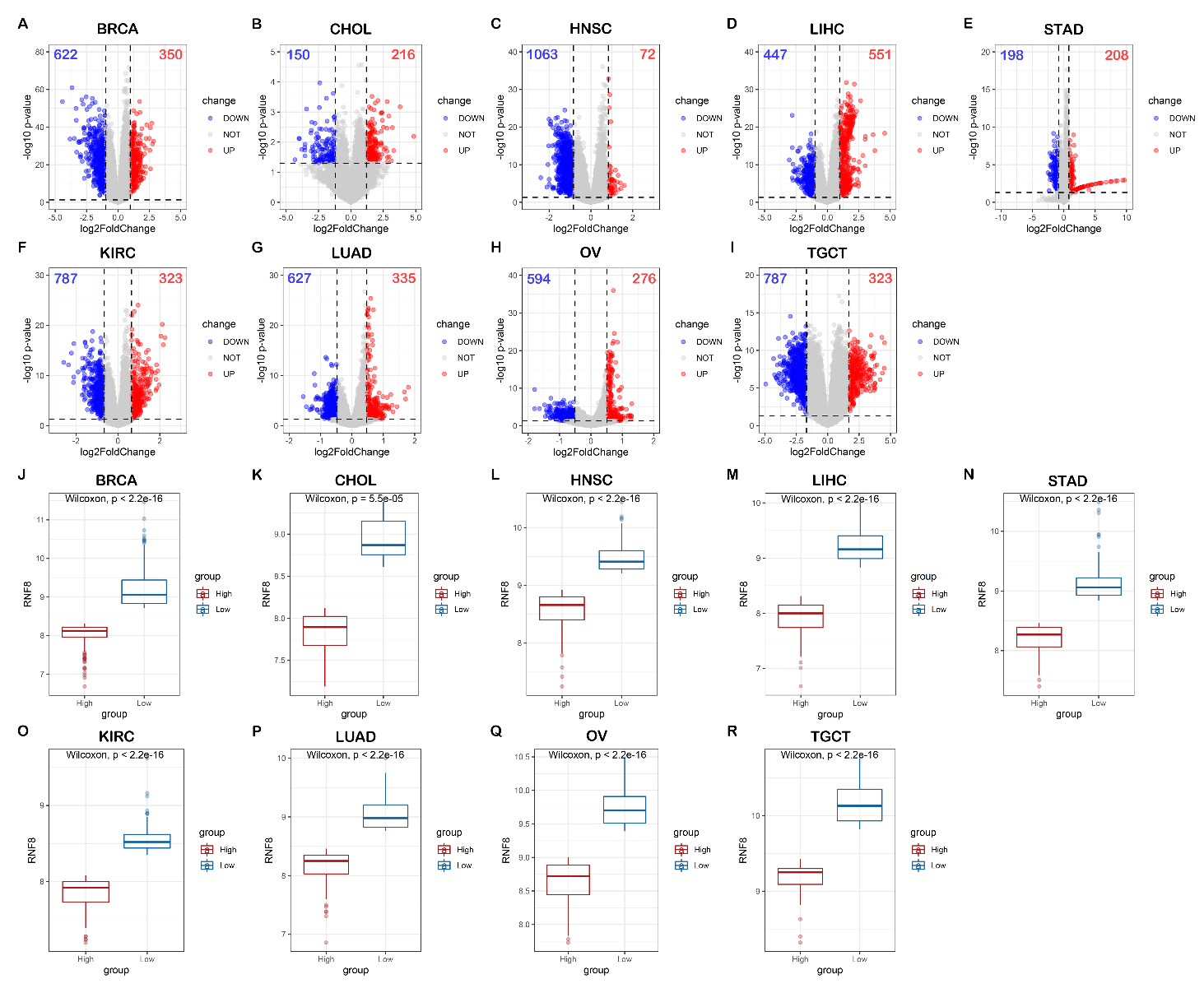
Figure S6. RNF8 abundance-based classification of cancer samples.**

**
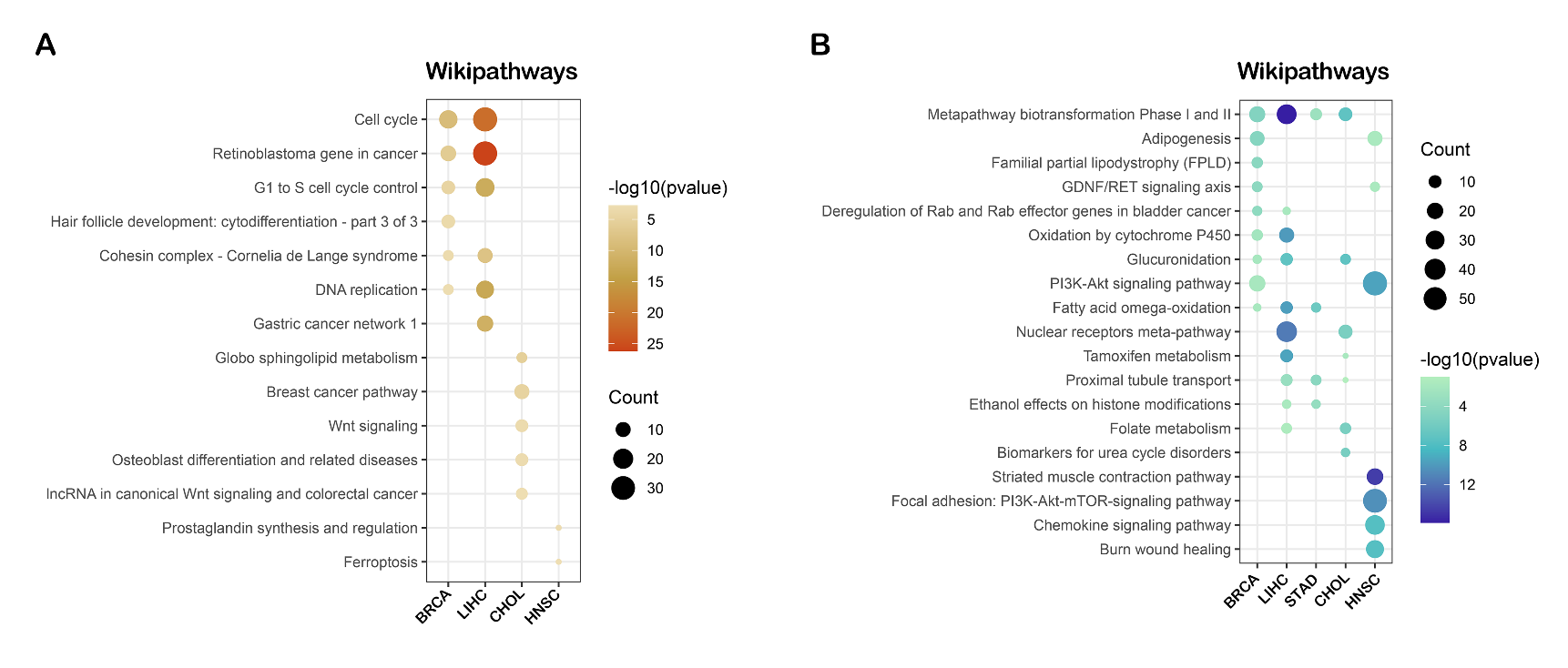
**

**Figure S7. Wikipathway analysis of RNF8 in cancers Group1.**

**
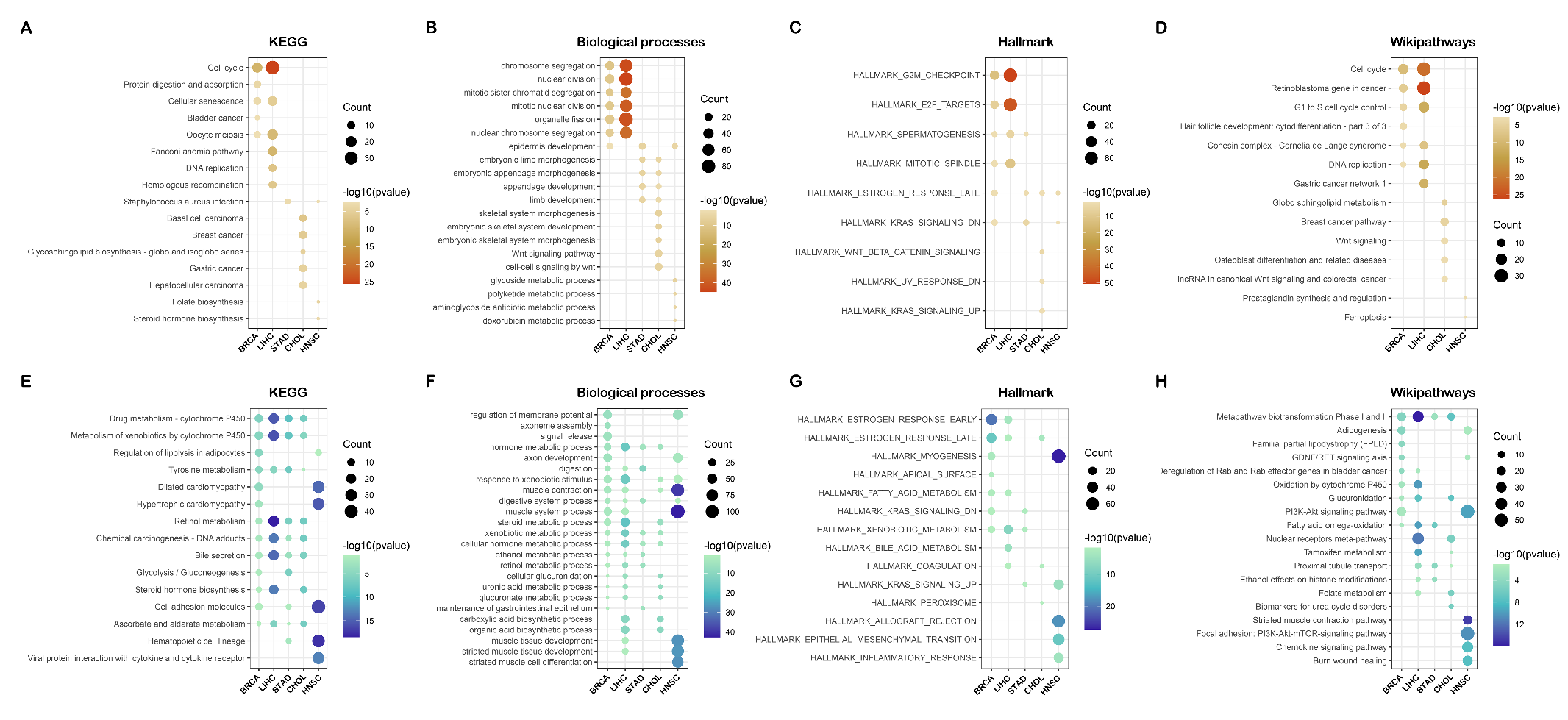
Figure S8. Functional analysis of RNF8 in cancers Group2.**


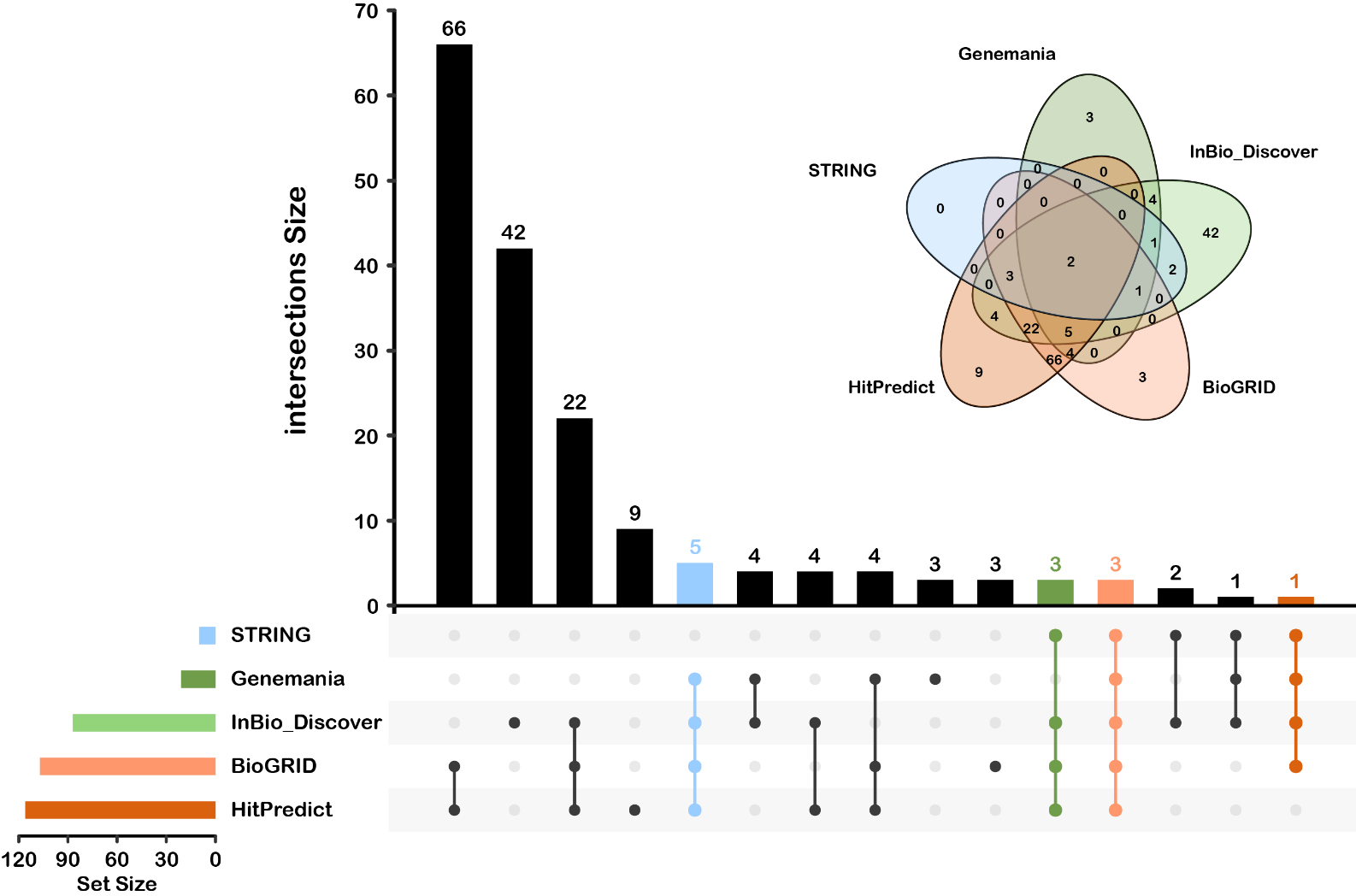
**Figure S9. Upsetplot and Venn diagrame showing the intersection between five PPI database-generated RNF8-interacting proteins.**


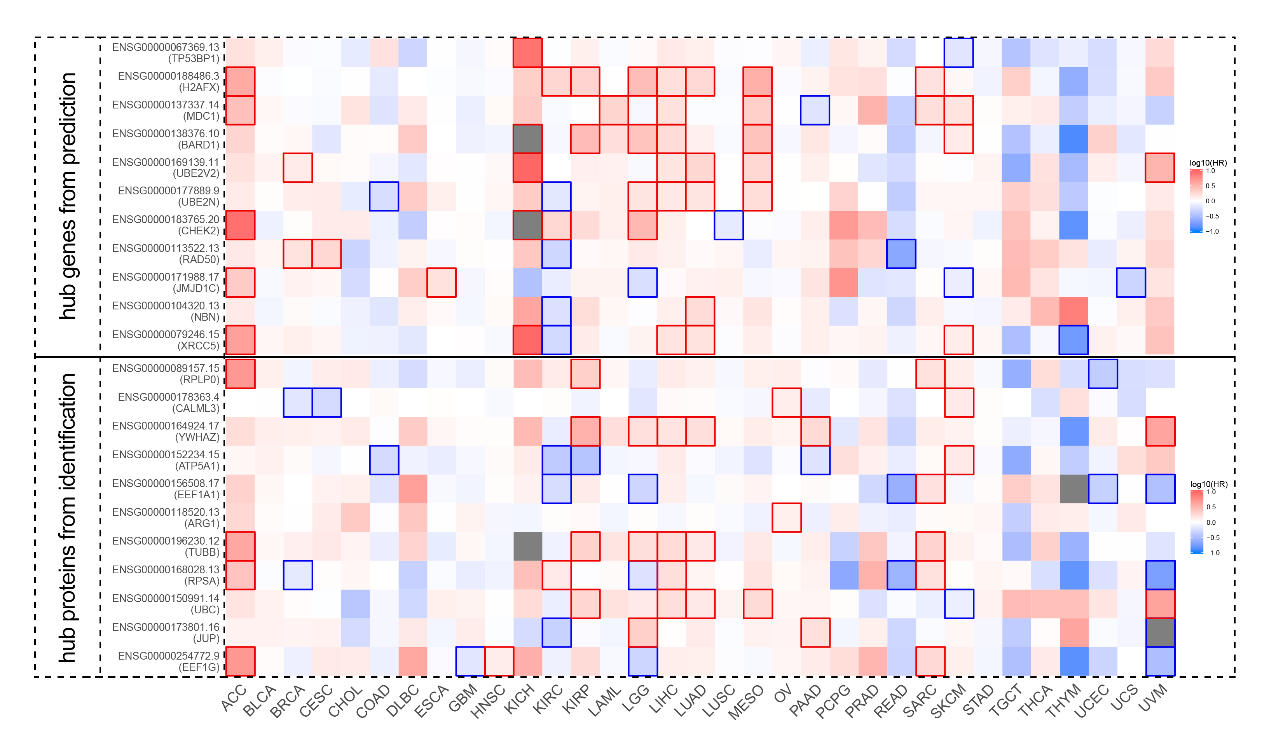
**Figure S10. The survival analysis of hub proteins.** The heatmap of the pan-cancer OS rate of 11 hub proteins by Kaplan-Meier survival analysis based on TCGA samples by GEPIA. A log rank p <0.05 was considered to indicate a statistically significant difference and are framed in red (positively correlated) or blue (negatively correlated)
