# Supplementary material for: A functional reference map of the RNF8 interactome in cancer
Source: Biol Direct. 2022 Jul 13;17:17. doi: 10.1186/s13062-022-00331-z (PMC9277853; doi:10.1186/s13062-022-00331-z)
Supplement: Supplementary file 15 — Additional file 15. Table S1. 218 proteins identified by intersection of two dataset. [file 13062_2022_331_MOESM15_ESM.docx]

**Table S1. 218 proteins identified by intersection of two dataset**

| KRT1 | FUS | PBRM1 | KRT78 | ALDOA | PCNA | YWHAQ |
| --- | --- | --- | --- | --- | --- | --- |
| MAP1B | CSTA | HSPA9 | RPS3 | HNRNPD | PRPS1 | LRRC59 |
| KRT2 | DSC1 | ATP5B | PKP1 | TIMM50 | PPP1CB | MDH2 |
| KRT10 | HSPA8 | NCL | EEF1A1 | PCBP2 | HBA1 | POLDIP2 |
| KRT9 | DSP | UBC | TGM1 | PCBP1 | CAPZA1 | FRG1 |
| HIST1H1C | KRT80 | DDB1 | SBSN | NACA | WDR5 | RPSA |
| ALB | SPTAN1 | KRT6B | NFXL1 | HNRNPK | LDHB | HBB |
| KRT5 | SPTBN1 | RBM25 | ACTB | LANCL1 | WDR82 | GNB2L1 |
| YBX1 | SF3B3 | PARP1 | SERPINB12 | AZGP1 | FBL | DIMT1 |
| KRT16 | HIST1H4A | KRT17 | PRDX2 | BUB3 | PYCR2 | GNB1 |
| KRT14 | HSPA1A | HSPA4 | THOC1 | RPLP0 | CDK1 | SLC25A3 |
| KRT6A | MAP4 | JUP | VPS35 | RFC3 | DNAJC9 | CLTA |
| DCD | CLTC | SF3B1 | AHSG | EIF2S1 | THOC6 | EIF3L |
| HNRNPU | KRT77 | DSG1 | MARCKS | DNAJB11 | ENO1 | SSR1 |
| LYZ | GAPDH | FLG2 | NPM1 | G3BP1 | TUBA1A | SERPINB3 |
| HRNR | LDHA | CAPRIN1 | EIF3I | ARG1 | ATP5C1 | RPL19 |
| MDN1 | EIF5B | KPRP | RFC4 | RPL7 | YWHAE | TUBB |
| RPL29 | HSPA5 | ABCF1 | EEF1D | QPCTL | EEF1B2 | RPS4X |
| EWSR1 | SERBP1 | S100A8 | DAP3 | EBNA1BP2 | PBDC1 | EIF4A2 |
| IGKV2D | ANXA2 | LCN1 | STRAP | RPS3A | AIMP1 | CTRL |
| GTF2H3 | AP2A1 | LGALS7 | H3F3B | PKM | SFN | PHB2 |
| RPS9 | LMNA | TPM1 | ASAH1 | HSP90AA1 | ATP5A1 | LCN2 |
| EEF1G | CALML5 | PSMA7 | NDUFS3 | PRDX1 | CTSD | CAT |
| RBBP4 | CALML3 | PHB | CPA4 | TUBA1B | FABP5 | PSMB1 |
| IGJ | FLG | TGM3 | APOD | P4HB | GGCT | PSMA5 |
| RFC5 | HSPB1 | ANXA5 | HADHB | EEF2 | S100A9 | ACTN1 |
| LTF | PABPC1 | A2ML1 | PSMB4 | YWHAZ | CKAP4 | IGHG1 |
| DDX47 | RUVBL2 | TRIM21 | EIF6 | HSP90B1 | ANXA4 | VDAC1 |
| EIF2S2 | CCT5 | ACTL6A | TUBB4B | AP2B1 | TPI1 | MTDH |
| OLA1 | DDOST | KRT3 | EIF2S3 | ANXA1 | CASP14 | S100A7 |
| LRPPRC | CTSB | RPL22 | SFPQ | CASP12 | PSMA6 | GSTP1 |
| TXN |  |  |  |  |  |  |
