# Supplementary material for: A functional reference map of the RNF8 interactome in cancer
Source: Biol Direct. 2022 Jul 13;17:17. doi: 10.1186/s13062-022-00331-z (PMC9277853; doi:10.1186/s13062-022-00331-z)
Supplement: Supplementary file 16 — Additional file 16. Table S2. Hub genes list identified from online tools and LC-MS-based identification. [file 13062_2022_331_MOESM16_ESM.docx]

**Table S2. Hub genes list from online tools and LC-MS-based identification**

| Hub genes from Online tools | Hub genes from identification |
| --- | --- |
| XRCC5 | EEF1G |
| NBN | JUP |
| JMJD1C | UBC |
| RAD50 | RPSA |
| CHEK2 | TUBB |
| UBE2N | ARG1 |
| UBE2V2 | EEF1A1 |
| BARD1 | ATP5F1A |
| MDC1 | YWHAZ |
| H2AX | CALML3 |
| TP53BP1 | RPLP0 |
